# Self‐Templated Hierarchically Porous Graphitic Aerogels for Emi Shielding

**DOI:** 10.1002/smll.202514369

**Published:** 2026-02-15

**Authors:** M. Shaharyar Wani, Yusuf O. Jimoh, Erick Zaragoza, Paul R. Prucnal, Craig B. Arnold

**Affiliations:** ^1^ Department of Mechanical & Aerospace Engineering Princeton University Princeton New Jersey USA; ^2^ Princeton Materials Institute Princeton University Princeton New Jersey USA; ^3^ Department of Electrical & Computer Engineering Princeton University Princeton New Jersey USA

**Keywords:** carbon aerogel, EMI shielding, hierarchical porosity, protein pyrolysis, tunable microstructure

## Abstract

As electronic systems become increasingly integrated into daily life, the demand for lightweight, effective, and environmentally sustainable materials for electromagnetic interference (EMI) shielding continues to grow. In this study, we investigate the EMI shielding performance of hierarchically porous graphitic aerogels (HGAs) synthesized from albumen protein through controlled pyrolysis. These single‐component, bio‐derived aerogels differ from conventional carbon aerogels by combining ultralow density, hierarchical porosity, and tunable electrical conductivity without templating, chemical activation, surface functionalization, or multistep processing. By varying the carbonization temperature, heating rate, and sample thickness, we demonstrate direct control over the aerogel's microstructure, density, and electrical properties, and consequently, the EMI shielding behavior. The HGA exhibits an outstanding specific shielding effectiveness of over 16 200 dB cm^2^ g^−^^1^, outperforming previously reported single‐component carbon‐based aerogels synthesized through simple pyrolysis by more than an order of magnitude. Furthermore, we show that processing conditions can be used to deliberately control the dominant attenuation mechanism, enabling a shift from reflection‐dominated to absorption‐dominated shielding behavior, with the latter being particularly promising for mitigating secondary electromagnetic pollution. These findings establish protein‐derived, single‐component HGAs as a simple, tunable, and high‐performance platform for EMI shielding, with broad implications for aerospace, electronics, and future wireless communication technologies.

## Graphical abstract

This work presents a self‐templated, single‐component hierarchically porous graphitic aerogel derived from albumen through pyrolysis for lightweight EMI shielding applications. Controlled thermal processing enables tunable structure, electrical conductivity, and attenuation mechanisms. The resulting ultralight aerogels exhibit exceptionally high specific shielding efficiency, offering a sustainable platform for absorption‐dominated EMI shielding.

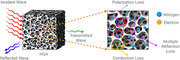

## 1 Introduction

As electronic systems become increasingly embedded in the technologies that shape modern life, the problem of electromagnetic interference has intensified. Devices used in satellite communications, radar, autonomous navigation, and wearable electronics operate in environments crowded with electromagnetic signals, where interference can disrupt functionality, degrade performance, and in some cases, pose risks to human health and environmental safety [1, 2, 3]. This is especially critical in the X‐band frequency range from 8 to 12 gigahertz, a part of the spectrum vital to defense, aerospace, and high‐speed communication systems [1]. Addressing this challenge calls for materials that not only attenuate electromagnetic waves effectively but are also low‐density, simple to process, thermally and chemically stable, and resilient under harsh environmental conditions.

Conventional electromagnetic shielding materials, such as metals and metal oxide composites, primarily attenuate incident waves through reflection due to their high electrical conductivity [4, 5, 6, 7, 8]. While they offer strong shielding performance, these materials present several key limitations; for instance, their high density makes them unsuitable for weight‐sensitive applications, and their rigidity and susceptibility to corrosion reduce long‐term reliability in harsh environments. More importantly, reflection‐based shielding can redirect electromagnetic waves back into the environment, contributing to secondary interference and complicating the electromagnetic landscape [2, 8, 9]. These drawbacks have led to growing interest in alternative materials that attenuate electromagnetic radiation primarily through absorption, offering a more efficient, practical, and environmentally compatible approach to shielding.

Carbon aerogels are uniquely suited to be versatile platforms for developing lightweight, multifunctional materials tailored to advance next‐generation electromagnetic shielding technologies. These 3D porous structures combine ultralow density with high electrical conductivity, thermal stability, and chemical robustness [10, 11, 12, 13, 14, 15, 16]. Their hierarchical pore structure, ranging from the nanometer to micrometer scale, promotes multiple electromagnetic wave attenuation mechanisms, including conductive loss, dielectric polarization, and internal scattering [17]. Beyond intrinsic structural advantages, their properties can be tuned through precursor selection, processing conditions, and post‐synthetic modifications [18]. Surface functionalization, in particular, offers a powerful means to tailor interfacial polarization and charge distribution, further enhancing shielding efficiency [19]. This level of tunability enables carbon aerogels to be adapted for a wide range of advanced applications, including energy storage, chemical sensing, catalysis, and thermal management [10, 11, 12, 18, 19, 20, 21, 22]. Taken together, these attributes establish carbon aerogels as an attractive and highly adaptable class of materials for overcoming the limitations of traditional metal‐based EMI shielding systems.

Recent studies have established bio‐based carbon aerogels as viable, environmentally sustainable alternatives for EMI shielding applications, and these comprise both single‐component carbons and multicomponent composite architectures [18]. Biopolymer‐based precursors such as cellulose, chitin, and chitosan are particularly attractive due to their abundance, structural versatility, and heteroatom‐rich chemistry. In general, the high EMI shielding performance is achieved in composite carbon‐based aerogel systems, where multiple constituents are integrated to enhance electrical conductivity and interfacial polarization. For example, chitin‐ and chitosan‐based composite carbon aerogels have reported specific shielding effectiveness (SSE) values exceeding 4 800 dB·cm^2^·g^−^^1^ [2], while wood‐derived carbons modified with zeolitic imidazolate frameworks (ZIFs) have shown SSE values as high as 11,330 dB·cm^2^·g^−^^1^ [9]. Some composite systems have even reported SSE values exceeding 19 000 dB·cm^2^·g^−^^1^ and achieve the high total attenuation through reflection mechanisms [23, 24]. While these approaches present significant progress, they often involve multiple processing steps, chemical templating, or activating agents, which introduce complexity and potentially reduce the scalability of these approaches. These constraints motivate the search for simpler, more sustainable fabrication methods that can match or surpass the EMI shielding performance of current state‐of‐the‐art systems.

In this study, we report the first demonstration of a single‐component ultra‐lightweight, hierarchically porous graphitic aerogel (HGA) synthesized through controlled pyrolysis of albumen protein for EMI shielding applications. Unlike conventional methods that rely on templating, chemical activation, or multistep processing, the present HGA forms through a self‐templated mechanism driven by the intrinsic foaming and carbonization behavior of albumen during pyrolysis. This facile approach yields monolithic aerogels with tunable density and electrical conductivity by adjusting the peak pyrolysis temperature and heating rate. These single‐component bio‐derived HGAs exhibit an SSE of over 16 200 dB·cm^2^·g^−^^1^, surpassing previously reported single‐component carbon or graphene aerogels prepared through simple pyrolysis routes. Furthermore, we show that the peak carbonization temperature, heating rate, and geometric parameters such as sample thickness can be systematically controlled to tailor both the SSE and the dominant attenuation mechanism, with absorption‐dominated shielding being particularly desirable for minimizing secondary electromagnetic reflections while maintaining high mass‐specific efficiency. These results establish a scalable and sustainable materials platform for high‐performance EMI shielding and demonstrate the potential of protein‐derived graphitic architectures as multifunctional electromagnetic materials.

## 2 Results and Discussion

HGAs were synthesized through the pyrolysis of albumen, a protein‐based precursor [12]. The synthesis begins with the lyophilization of the albumen precursor to remove solvent and obtain a dry precursor. This dried material is then subjected to pyrolysis under an inert nitrogen atmosphere, where the thermal treatment parameters are carefully controlled. Among these, the peak pyrolysis temperature or peak carbonization temperature, defined as the highest temperature reached during the process, plays a critical role in determining the structural and chemical evolution of the material. It influences the decomposition behavior of the protein, drives molecular rearrangement, and promotes the development of graphitic domains [12, 15, 16]. As a result, the peak temperature has a direct impact on the microstructure, elemental composition, and functional properties of the resulting aerogel.

**FIGURE 1:**
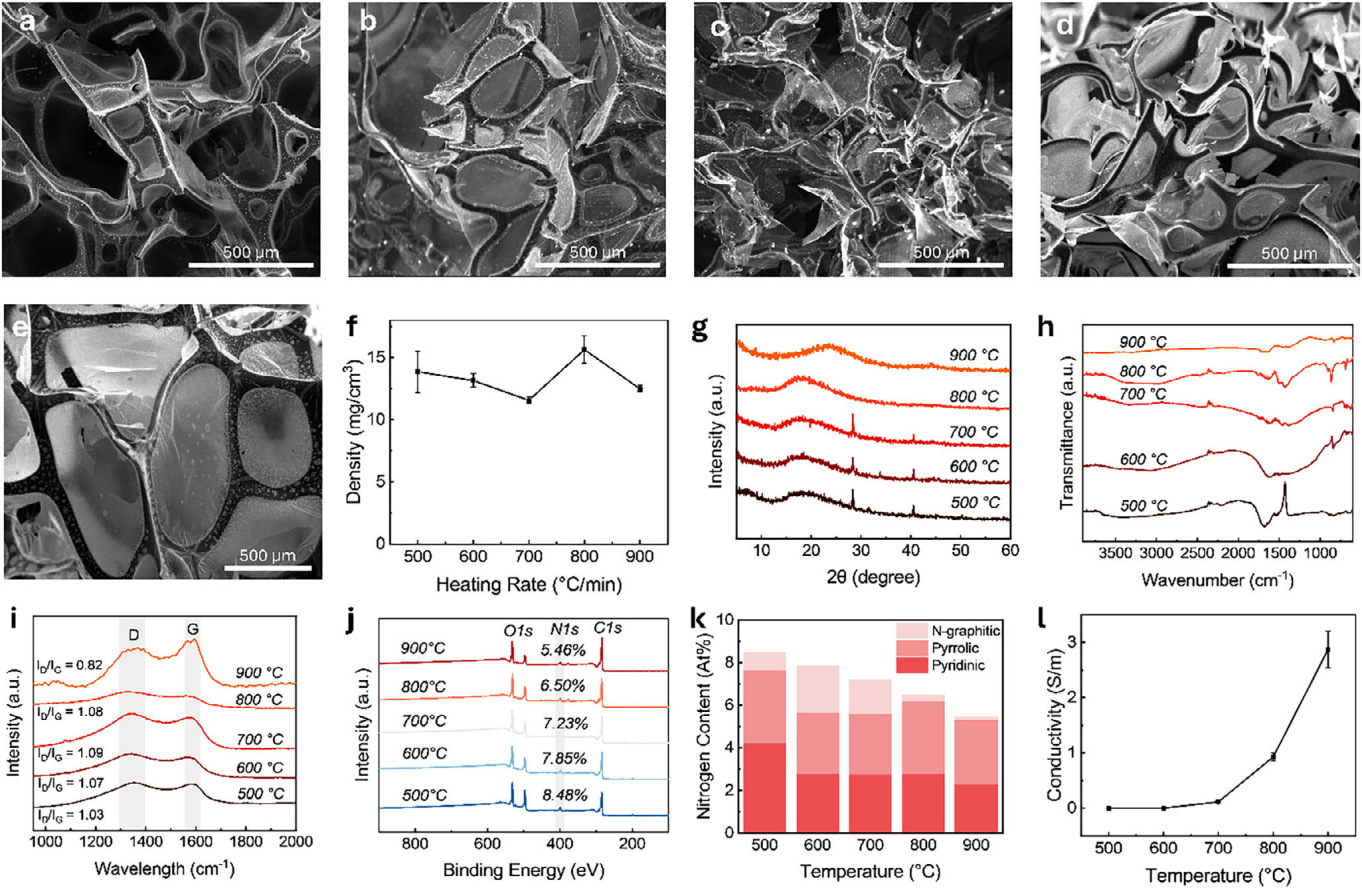
SEM micrographs of HGA samples prepared at peak processing temperatures of (a) 500, (b) 600, (c) 700, (d) 800, and (e) 900°C. (f) Density of the corresponding samples. (g) XRD patterns show phase evolution across different peak processing temperatures. (h) FTIR spectra indicating changes in functional groups as a function of peak processing temperature. (i) Raman spectra demonstrating graphitic ordering trends as a function of temperature. (j) XPS survey scan showing compositional variations for samples prepared at different peak temperatures and (k) showing nitrogen composition for corresponding samples. (l) Electrical conductivity trend as a function of peak processing temperature.

Figure 1a–e present scanning electron microscopy (SEM) images of HGAs prepared at peak pyrolysis temperatures of 500, 600, 700, 800, and 900°C, with the heating rate fixed at 5°C/min and a residence time of 4 h. All samples exhibit monolithic architecture comprising interconnected sheet and fiber‐like morphologies. This structural evolution arises from a thermally induced self‐assembly process during pyrolysis [15, 16]. As the temperature approaches ∼ 250°C, the albumen precursor undergoes thermal softening and transitions into a viscous intermediate. With further heating, thermal decomposition initiates the evolution of gases, leading to foaming within the intermediate. Continued gas release expands the foam, redistributing material toward the cell walls due to surface tension effects. This results in a stable polyhedral framework with thin carbonaceous sheets and fiber‐like connections. The aerogels obtained are ultralight monoliths composed predominantly of air, exhibiting bulk densities in the range of 11.56 to 15.64 mg cm^−^^3^ (Figure 1f). Samples from at least four independent batches were analyzed to ensure consistency and reproducibility, and the reported values represent averaged densities with only small variations across batches. The observed reproducibility arises from the intrinsic self‐foaming behavior of the protein precursor, which eliminates the need for external foaming agents or sacrificial templates. This inherent mechanism ensures uniform foaming throughout the material and effectively overcomes the scalability and reproducibility challenges encountered in conventional methods, where inconsistent dispersion of external templates or foaming additives often limits process reliability.

The peak pyrolysis temperature strongly influences the structural evolution of the carbon framework in the synthesized aerogels. XRD patterns of samples treated at different peak temperatures are shown in Figure 1g. The sample processed at 500°C exhibits a broad, low‐intensity peak centered near 18°, indicative of an amorphous carbonaceous phase typically associated with char formation during incomplete carbonization [25]. As the peak temperature increases to 600°C, 700°C, and 800°C, the intensity and sharpness of the (002) diffraction peak become progressively more pronounced. This evolution suggests the development of turbostratic carbon and the growth of larger aromatic domains through thermal rearrangement and removal of functional groups [25]. At 900°C, the (002) peak shifts toward higher angles, approaching 26°, which is consistent with the (002) reflection of graphitic carbon. This shift indicates a decrease in interlayer spacing and improved ordering of carbon layers [25, 26]. Additionally, a secondary peak appears near 44° for the 900°C sample, corresponding to the (100) in‐plane reflection, further confirming the formation of graphitic structures [25]. These observations collectively indicate a transition from highly amorphous carbon to a relatively ordered, graphitic structure with increasing thermal treatment.

Figure 1h shows the FTIR spectra of HGA samples prepared at different peak pyrolysis temperatures. The sample treated at 500°C displays several prominent absorption bands, including a broad peak around 3 300 cm^−^^1^ corresponding to O─H and N─H stretching vibrations, as well as peaks at approximately 1 635 and 1 513 cm^−^^1^, associated with the amide I (C═O stretching) and amide II (C─N stretching and N─H bending) modes, respectively [16]. These features are typical of protein‐based materials and indicate the presence of organic functional groups derived from the albumen precursor. As the peak pyrolysis temperature increases to 600, 700, 800, and 900°C, the intensity of these bands gradually decreases. This trend reflects the thermal decomposition and removal of functional groups such as hydroxyl, amide, and alkyl species as the material undergoes carbonization [16]. At 900°C, most characteristic peaks disappear, suggesting the formation of a highly carbonaceous material with minimal IR‐active functionality, consistent with progressive chemical transformation and loss of heteroatoms during pyrolysis.

Figure 1i shows the Raman spectra of HGA samples prepared at different peak pyrolysis temperatures, highlighting the evolution of structural ordering in the carbon framework. All samples exhibit two characteristic peaks centered around 1346 cm^−^^1^ (D band) and 1,585 cm^−^^1^ (G band). The D band is associated with the A_1_g breathing mode of disordered sp^2^ carbon rings, while the G band arises from the E_2_g in‐plane vibrational mode of sp^2^‐hybridized carbon atoms in the material [12, 16]. The intensity ratio of these bands (I_D_/I_G_) provides a quantitative measure of defect density and the degree of graphitic ordering. As the peak pyrolysis temperature increases from 500°C to 900°C, the I_D_/I_G_ ratio decreases from 1.03 to 0.82. This trend displays a progressive reduction in disorder and growth of ordered graphitic domains with increasing temperature [2, 16]. These results are consistent with the XRD data (Figure 1g), which show sharpening and shifting of the (002) peak toward 26°, indicating improved layer stacking and interlayer ordering. The FTIR spectra (Figure 1h) further support this observation, showing the gradual disappearance of functional groups such as O─H, N─H, and C═O, which are typically removed during high‐temperature carbonization. While higher temperatures promote graphitic ordering through the elimination of heteroatoms and restructuring of the carbon matrix, they may also induce localized defects or voids due to the release of volatile species [2, 16]. Nonetheless, the overall decrease in the I_D_/I_G_ ratio suggests an increase in structural ordering, leading to the formation of a relatively more crystalline HGA.

X‐ray photoelectron spectroscopy (XPS) analysis was conducted to investigate the elemental composition of the HGAs and to understand the influence of peak pyrolysis temperature on chemistry. Figure 1j presents the survey spectra of samples synthesized at different temperatures. All spectra show prominent peaks at approximately 284 eV (C 1s), 399 eV (N 1s), and 531 eV (O 1s), indicating the presence of carbon, nitrogen, and oxygen in each sample [2]. Quantitative analysis shows that nitrogen content decreases from 8.48 atomic percent at 500°C to 5.46 atomic percent at 900°C (Figure 1j; Table S1). This decline is attributed to the gradual removal of nitrogen‐containing groups through thermal decomposition, which likely results in the release of nitrogen in the form of small gaseous species [2]. The overall decrease in nitrogen content with increasing temperature is consistent with the progressive de‐functionalization observed in FTIR and the increased graphitic ordering indicated by Raman spectroscopy. Despite this reduction, a measurable amount of nitrogen remains even at the highest carbonization temperature, suggesting partial retention of nitrogen functionalities within the carbon framework.

To further probe the nitrogen chemical environment, the N1s peak was deconvoluted into three components corresponding to pyridinic, pyrrolic, and graphitic nitrogen (Figure 1k; Figure S1) [20]. At 500°C, the nitrogen is predominantly present in pyridinic (49.9%) and pyrrolic (40.5%) forms, which are typically associated with edge and defect sites in disordered carbon. With increasing temperature, the fraction of graphitic nitrogen increases, reaching a maximum of 27.8% at 600°C, indicating incorporation of nitrogen into the conjugated sp^2^ carbon lattice during early carbonization. However, at 800 and 900°C, the graphitic nitrogen content decreases significantly, while pyrrolic nitrogen becomes dominant (52.5% and 55.1%, respectively). This shift suggests that, although nitrogen is retained, its incorporation into graphitic domains becomes thermodynamically unfavorable at higher temperatures, possibly due to atom rearrangement or volatilization during extended heat treatment. Since nitrogen doping can modify the electronic structure of carbon by introducing local charge variations and active electronic sites, the nitrogen content and bonding environment may influence both the electrical conductivity and electromagnetic response of the aerogels [2, 27].

Electrical conductivity is an important parameter in evaluating the potential of carbon‐based materials for applications like EMI shielding. In conductive materials, electromagnetic wave attenuation is often governed by reflection, which is strongly influenced by the availability of free charge carriers [2, 28]. In HGAs, the peak pyrolysis temperature strongly influences the formation of conductive pathways. Electrical conductivity was determined from measured resistance, with values averaged over at least four samples to ensure consistency and reproducibility. Figure 1l presents electrical conductivity as a function of peak pyrolysis temperature. Samples treated at 500°C and 600°C exhibit very low conductivities of 0.252 µS m^−^^1^ and 0.190 mS m^−^^1^, respectively. At these temperatures, the material remains largely disordered, consisting of amorphous char with limited structural ordering and poor electrical connectivity between carbon domains [16]. This is consistent with the broad XRD peak at low angles (Figure 1g), the high I_D_/I_G_ band intensity ratio in Raman spectra (Figure 1i), and the presence of relatively high oxygen‐ and nitrogen‐containing functional groups observed in FTIR and XPS analyses (Figure 1h,j).

As the pyrolysis temperature is increased to 700, 800, and 900°C, a substantial increase in electrical conductivity is observed, reaching 0.11, 0.93, and 2.87 S/m, respectively. This enhancement is attributed to the progressive carbonization and formatixon of relatively more ordered carbon networks. XRD results show sharpening and shifting of the (002) peak toward 26° (Figure 1g), while Raman spectra reveal a decreasing I_D_/I_G_ ratio (Figure 1i), both indicating increasing graphitization. FTIR and XPS analyses (Figure 1h,j) further confirm the removal of heteroatoms such as oxygen and nitrogen, which contribute to the development of a more conjugated and electronically delocalized carbon structure. At 800°C and 900°C, there is an increase in the electrical conductivity, which may result from the overall improvement in graphitic ordering and reduction in defect density [2]. These findings indicate that increasing the peak carbonization temperature enhances structural reordering, leading to the development of a more electrically conductive carbon network, which is suitable for applications like EMI shielding.

**FIGURE 2:**
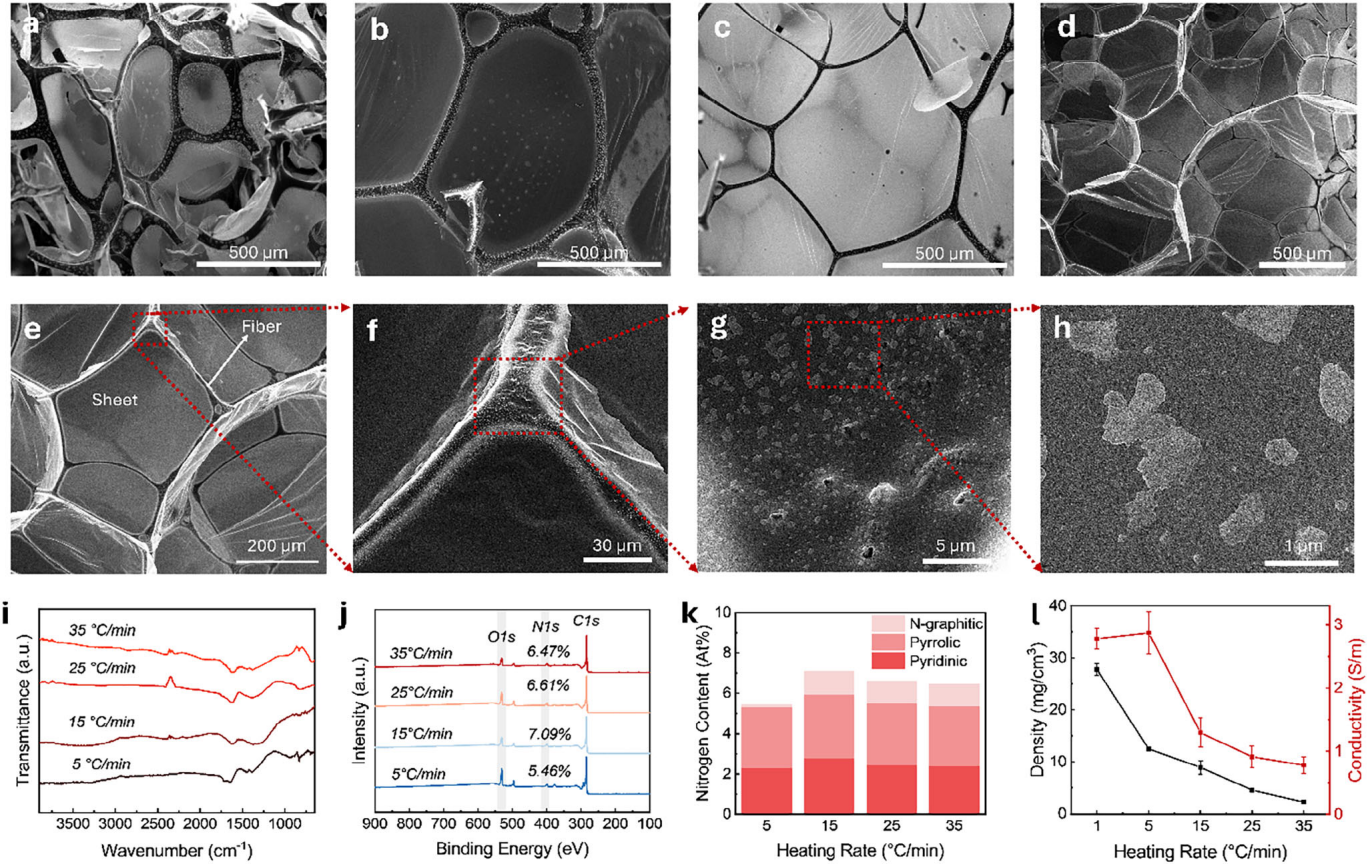
(a–d) SEM micrographs of HGA samples prepared at heating rates of 5, 15, 25, and 35°C/min, respectively, illustrating morphological changes influenced by heating rate. (e–h) High‐magnification SEM images showing sheet and fiber‐like morphologies with micropores and a rough surface. (i) FTIR spectra capturing trends in functional groups at different heating rates. (j) XPS survey scan depicting compositional variations, and (k) measured nitrogen content for each sample. (l) Density and electrical conductivity as a function of heating rate.

Heating rate is a critical parameter in the pyrolytic transformation of protein precursors into HGAs. It governs the rate of decomposition, timing of gas release, and viscoelastic behavior of the intermediate carbonaceous material, all of which contribute to the final structural morphology formation and porosity of the aerogel [16]. Faster heating can lead to rapid gas evolution and limited structural relaxation, while slower heating allows more time for molecular rearrangement, bubble coalescence, and network stabilization during the foaming stage of pyrolysis. To investigate these effects, albumen‐derived HGAs were synthesized at heating rates of 1, 5, 15, 25, and 35°C/min, with the peak pyrolysis temperature and residence time of 900°C and 4 h, respectively. SEM images of the resulting aerogels reveal a consistent overall morphology comprising interconnected sheets and fiber‐like structures across all samples (Figure 2a–d). However, clear differences in feature size and surface characteristics are observed as a function of heating rate. At slow heating rates (e.g., 5°C/min), the fiber‐like structures appear thicker, with broader nodes connecting extended sheet regions (Figure 2a). In contrast, faster heating rates (e.g., 35°C/min) produce finer fiber networks (Figure 2b–d). This morphological change is attributed to accelerated gas evolution and foam expansion at faster heating rates, which likely limit the time available for bubble coalescence and coarsening during the viscous intermediate stage. Higher magnification SEM images of HGA prepared at 35°C/min heating rate are shown in Figure 2e–h, further revealing that fiber surfaces exhibit roughness and the presence of nanopores, which may form due to gas release and deposition of material.

To quantitatively evaluate the effect of heating rate on HGA structure, the average width of fiber‐like structures was estimated from SEM images. As summarized in Figure S2, fiber width shows a decreasing trend with increasing heating rate, dropping from about 65 µm at 1°C/min to about 14 µm at 35°C/min. This trend aligns with previously reported behavior in pyrolyzed protein‐derived foams, where faster heating reduces the timescale for viscous flow and coarsening, leading to finer microstructures [16]. The observed heating rate‐dependent morphological variations are expected to impact HGA properties such as porosity, electrical conductivity, and electromagnetic wave attenuation. In particular, thinner and more interconnected networks at higher heating rates may enhance internal scattering pathways, which is important for EMI shielding applications as discussed in later sections.

Figure 2i presents the FTIR spectra of HGA samples prepared at different heating rates (5, 15, 25, and 35°C/min). Across all samples, the spectra are largely featureless, with a weak absorption band near 1635 cm^−^^1^; this peak corresponds to residual C═O or C═C stretching vibrations, possibly from conjugated aromatic domains [16]. The absence of peaks associated with O─H, N─H, or C─H stretching suggests that the organic functional groups present in the protein precursor have been extensively removed during pyrolysis. The similarity of the FTIR spectra across all heating rates indicates that, despite morphological differences, the overall de‐functionalization is comparable. This observation is consistent with previous findings, where samples pyrolyzed at the same peak temperature exhibited similar levels of graphitization, regardless of heating rate [16]. Since all samples undergo carbonization at 900°C, the elimination of heteroatom‐containing moieties appears to be governed primarily by the peak carbonization temperature rather than the rate of heating.

To investigate the influence of heating rate on nitrogen retention and bonding states in HGAs, XPS analysis was performed on samples pyrolyzed at 900°C using heating rates of 1, 5, 15, 25, and 35°C/min. The survey spectra confirm the presence of carbon (C 1s), nitrogen (N 1s), and oxygen (O 1s) in all samples (Figure 2j; Table S2). The nitrogen content shows moderate variation across the heating rates, ranging from 5.46 atomic percent at 5°C/min to 7.09 atomic percent at 15°C/min, then slightly decreasing to 6.61 and 6.47 atomic percent at 25 and 35°C/min, respectively. To further understand how heating rate affects nitrogen bonding, the high‐resolution N 1s spectra were deconvoluted into pyridinic, pyrrolic, and graphitic components (Figure 2k; Figure S3). All samples display a similar distribution dominated by pyridinic and pyrrolic nitrogen. The graphitic nitrogen fraction, though lower in magnitude, increases from 0.14 atomic percent at 5°C/min to ∼1.1 atomic percent across the higher heating rates. This increase suggests that faster heating may facilitate relatively higher retention of graphitic nitrogen, even under the same final carbonization temperature. Overall, these results suggest that heating rate has a subtler effect than peak temperature but can still impact nitrogen content and bonding states to some extent.

The heating rate during pyrolysis plays an important role in shaping the physical characteristics of HGAs, particularly their density, porosity, and structural morphology. Under an inert nitrogen atmosphere, intense thermal treatment leads to the decomposition of the albumen precursor, resulting in significant mass loss due to volatilization of organic species [16]. Simultaneously, gas evolution during decomposition promotes structural foaming and volumetric expansion, contributing to a highly porous architecture. Figure 2l (black curve) shows the measured bulk densities of HGAs synthesized at different heating rates. An inverse correlation is observed between heating rate and aerogel density. At the heating rate of 1°C/min, the aerogel exhibits a density of 27.8 mg/cm^3^, indicating limited expansion and gas trapping. As the heating rate increases, the density decreases progressively to 12.5, 8.9, and 4.6 mg/cm^3^ for 5, 15, and 25°C/min, respectively, reaching an ultralow value of 2.27 mg/cm^3^ at 35°C/min. Moreover, specific surface area and pore characteristics of the samples were evaluated from nitrogen adsorption–desorption isotherms (Figure S4a–e). Increasing the heating rate from 1 to 35°C min^−^^1^ resulted in more than 75‐fold rise in BET surface area, from 6.1 to 458.6 m^2^ g^−^^1^ (Figure S4f). The corresponding pore size distributions (Figure S4g) reveal a progressive shift toward smaller pores with increasing heating rate. This trend can be attributed to the kinetics of decomposition and gas evolution [16]. At slower heating rates, the precursor spends more time in intermediate temperature ranges, which may promote densification or collapse of evolving bubble‐like structures. In contrast, higher heating rates promote rapid decomposition and highly vigorous gas evolution, generating higher porosity and extensive foaming, ultimately producing very lightweight, porous networks. This behavior is consistent with the observed SEM morphologies showing increasingly thinner fibers and sheet‐like features with increasing heating rate.

The electrical conductivity of HGAs is strongly influenced by the heating rate during pyrolysis, which modulates the internal structure and connectivity of the carbon framework. Figure 2l (red curve) presents the electrical conductivity of HGAs synthesized at different heating rates. A decreasing trend is observed with increasing heating rate; the conductivity is highest for samples prepared at 1°C/min and 5°C/min (2.78 S/m and 2.87 S/m, respectively) and gradually decreases to 1.29, 0.91, and 0.78 S/m for heating rates of 15, 25, and 35°C/min. This trend is linked to the bulk density (Figure 2l, black curve) and porosity (Table S3) of the aerogels. Lower heating rates result in denser microstructures with thicker fiber‐like morphologies, supporting more efficient charge transport. In contrast, higher heating rates promote rapid gas evolution and expansion, resulting in highly porous and ultralight structures with reduced electrical conductivity.

The heating rate also has a significant influence on the mechanical properties of the developed aerogels, as described in our previous studies [12, 16]. As shown in Table T4, slower heating rates during pyrolysis produce denser networks with thicker fibers, resulting in higher compressive strength (>15 kPa) and greater structural rigidity. In contrast, faster heating rates yield more porous and lightweight frameworks with reduced strength but enhanced flexibility [16]. Moreover, compression loading‐unloading tests performed by Ozden et al. indicate that HGAs can withstand up to 90% strain without structural failure, confirming their excellent mechanical resilience and stability [12]. This tunable relationship between heating rate, density, and conductivity offers a means of tailoring HGA properties for applications like EMI shielding. For instance, lower heating rates may be favorable for applications requiring higher conductivity and wave reflection, while faster heating rates enable ultralow density materials with potentially enhanced scattering and absorption due to their increased porosity. The ability to modulate these properties through a simple processing parameter, such as heating rate, demonstrates the versatility of the pyrolytic approach to designing multifunctional HGAs.

**FIGURE 3:**
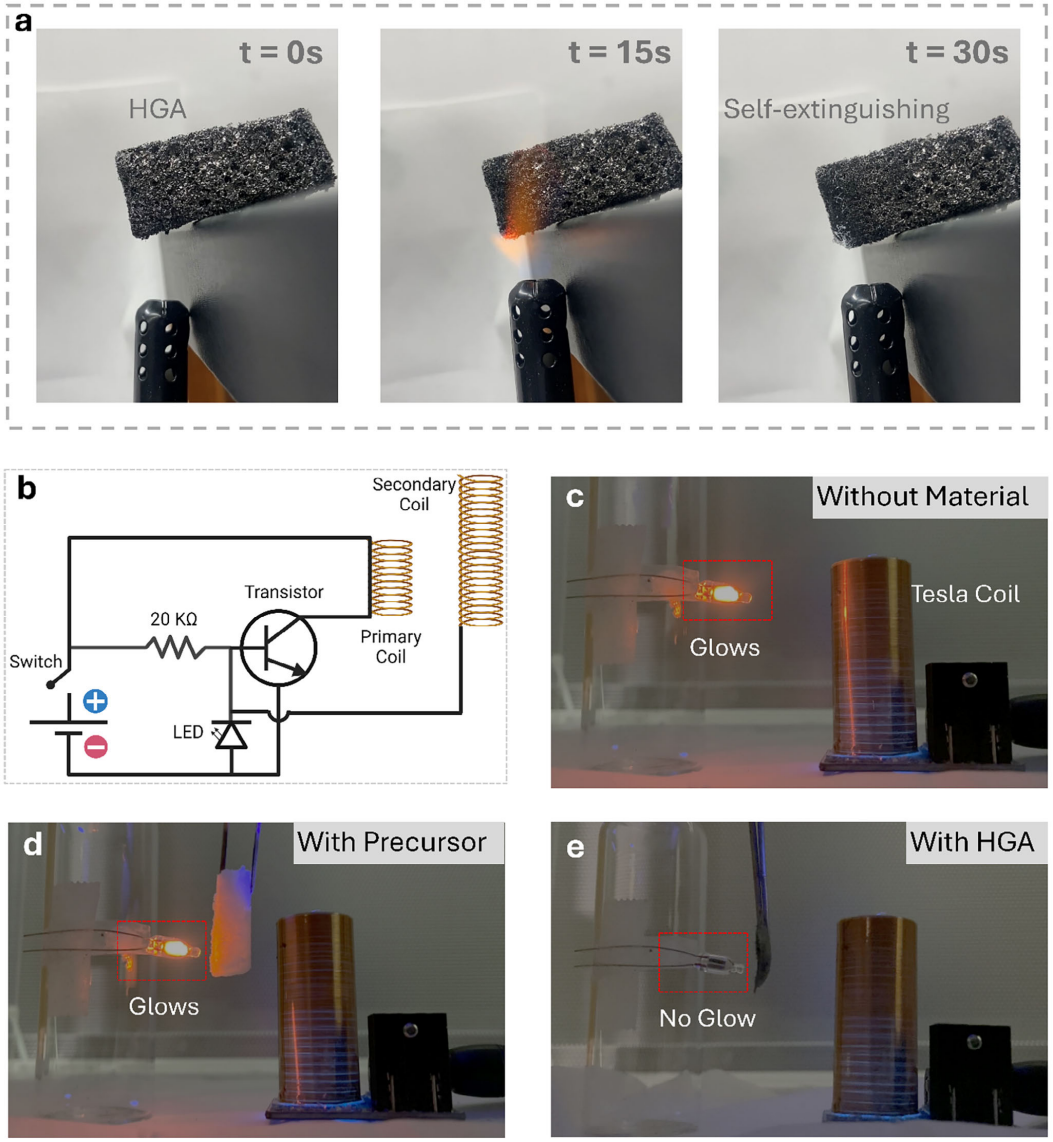
(a) Photographs of the combustion behavior of HGA under oxidative conditions. (b) Schematic of the Tesla coil circuit used to demonstrate EM wave shielding. (c–e) Digital photographs illustrating the effective EM shielding performance of the HGA.

The thermal stability of HGAs is a critical factor for their use in high‐temperature and oxidative environments. To evaluate this, combustion testing and thermogravimetric analysis (TGA) were performed (Figure 3a; Figure S5). When exposed to an open flame for 30 s, the HGA becomes red‐hot without producing smoke and immediately self‐extinguishes once the flame is removed, demonstrating good resistance to combustion. The TGA curve further shows a minor mass loss of approximately 6% at 400°C, consistent with values reported for similar aerogels [29]. This small loss is primarily attributed to the desorption of adsorbed moisture and the release of trapped gases. Moreover, these bio‐derived aerogels exhibit good stability under moisture‐rich conditions and have been previously demonstrated for microplastic removal and desalination, maintaining their structural integrity and functional performance over 50 operational cycles [12]. This stability of HGA arises from the high degree of carbonization and the formation of graphitic domains within the HGAs, as evidenced by the Raman analysis (Figure 1i), which impart intrinsic resistance to moisture, oxidation, and thermal degradation.

To demonstrate the electromagnetic shielding effectiveness of the HGA, a qualitative test was performed using a Tesla coil setup, which generates an alternating electromagnetic field. The setup consists of a bulb placed within the electromagnetic induction zone of the Tesla coil (Figure 3b). In the absence of the shielding material (Figure 3c), the bulb visibly glows due to induction from the surrounding electromagnetic field. When a protein precursor sample is introduced between the Tesla coil and the bulb (Figure 3d), the bulb continues to glow, indicating that the uncarbonized material does not significantly attenuate the electromagnetic field. In contrast, when an HGA sample carbonized at 900°C is introduced (Figure 3e), the bulb ceases to light up, demonstrating substantial attenuation of the incident electromagnetic field. The suppression of electromagnetic induction in the presence of the HGA indicates that the material acts as an effective barrier to EM waves. While this setup does not yield quantitative values, it offers a visual demonstration of the shielding ability of HGA.

**FIGURE 4:**
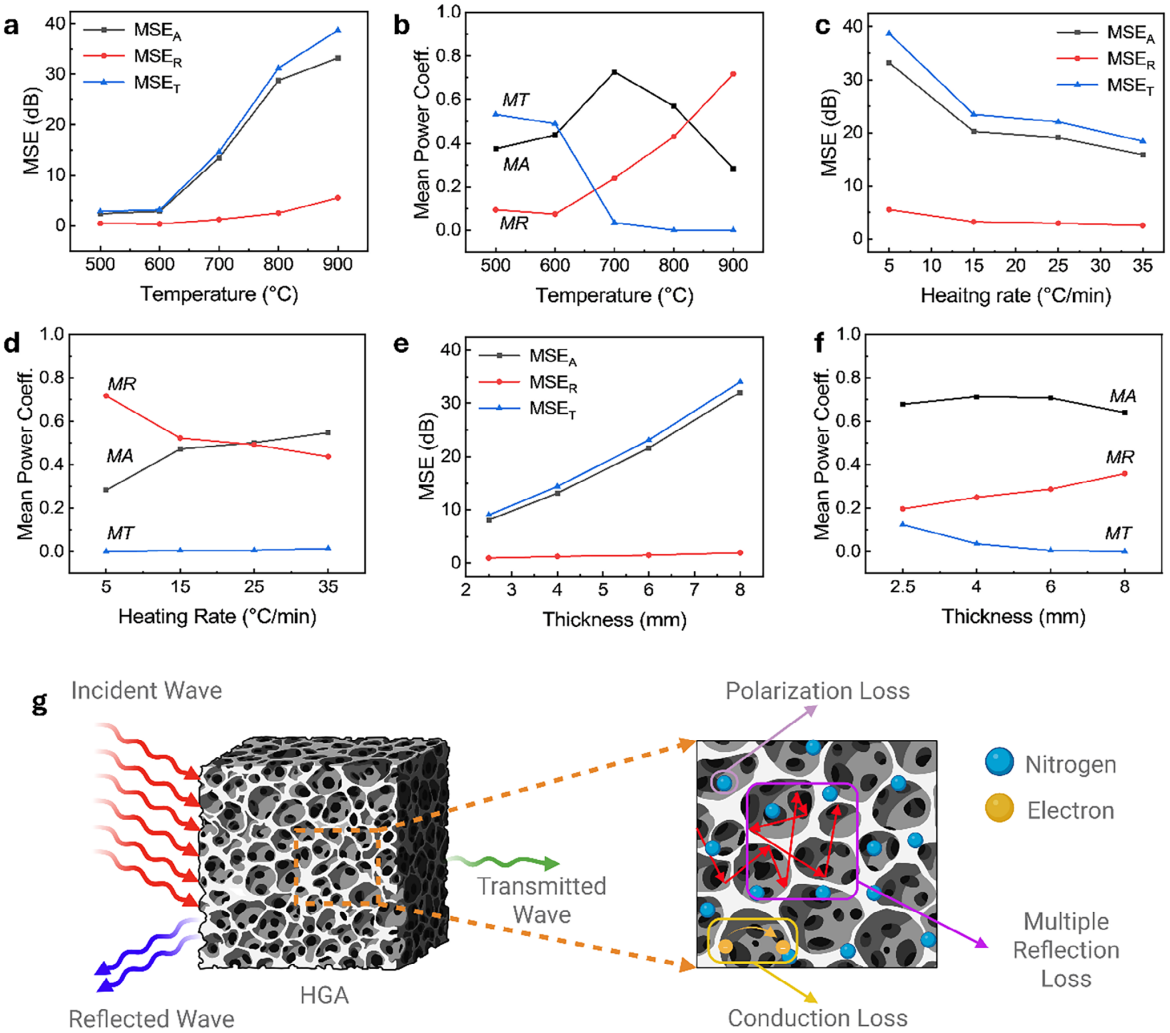
MSE and power coefficients of HGA samples prepared under different processing conditions. (a) MSE of samples carbonized at 500, 600, 700, 800, and 900°C, with a constant heating rate of 5°C min^−^^1^ and a thickness of 5 mm. (b) Corresponding mean power coefficients including absorption (MA), reflection (MR), and transmission (MT) for samples prepared at different carbonization temperatures. (c) MSE of samples synthesized at heating rates of 5, 15, 25, and 35°C min^−^^1^, with a constant peak processing temperature of 900°C and a thickness of 5 mm. (d) Corresponding mean power coefficients for samples prepared at different heating rates. (e) MSE of samples with thicknesses of 2.5, 4, 6, and 8 mm, prepared at a heating rate of 35°C min^−^^1^ and a peak processing temperature of 900°C. (f) Corresponding mean power coefficients for samples with different thicknesses. (g) Schematic illustration of electromagnetic wave interactions within the HGA structure.

To systematically evaluate the EMI shielding performance of the HGAs and elucidate the underlying attenuation mechanisms, measurements were conducted using a waveguide setup coupled with a vector network analyzer (Figure S6). The samples carbonized at peak pyrolysis temperatures ranging from 500 to 900°C at a heating rate of 5°C/min were tested in the X‐band frequency range (8–12 GHz), with each specimen having a thickness of 5 mm. Figure 4a shows the mean shielding effectiveness (MSE) of samples prepared with different peak pyrolysis temperatures, indicating improved attenuation properties with progressive carbonization. The sample prepared at 500°C exhibits a minimal total mean shielding effectiveness (MSE_T_) of 2.78 dB, consistent with its low electrical conductivity and partially carbonized structure. At 600°C, the shielding slightly improves to 3.14 dB. A significant enhancement is observed at 700°C, where MSE_T_ rises to 14.62 dB, which may be attributed to improved carbonization and consequent rise in electrical connectivity. Samples treated at 800°C and 900°C show pronounced improvements, with MSE_T_ values of 31.22 and 38.73 dB, respectively. These values correspond to more than 99.9% attenuation of incident electromagnetic waves and reflect the role of enhanced graphitization (Figure 1i) and increased electrical conductivity (Figure 1l) with increasing carbonization temperature. Figure 4a further presents the breakdown of attenuation into mean absorption shielding effectiveness (MSE_A_) and mean reflection shielding effectiveness (MSE_R_) components. MSE_A_ increases significantly from 2.35 dB at 500°C to 33.22 dB at 900°C, while SE_R_ increases from 0.42 to 5.51 dB across the same range. Figure S7a–c presents the shielding effectiveness (SE) of all samples as a function of frequency, showing that SE remains nearly constant across the X‐band (8–12 GHz), indicating frequency‐independent shielding performance. Evidently, these results indicate that both absorption and reflection improve with increasing carbonization temperature.

Identifying whether reflection or absorption is the dominant attenuation mechanism is an important consideration in the design of effective EMI shielding materials. From an application perspective, materials that primarily attenuate electromagnetic radiation through absorption are particularly attractive because they minimize secondary radiation and reduce stray signal interference, which is especially relevant in environments such as aerospace and advanced communication systems [17, 18, 19, 28, 30]. While the MSE metric gives a way to quantify relative shielding properties of the material, it does not provide an absolute comparison of the individual contributions of reflection, absorption, and transmission, and therefore, it is often misinterpreted [4]. For instance, MSE_A_ gives information about the fraction of the non‐reflected wave (rather than the total incident wave) that is absorbed, MSE_R_ gives a measure of the fraction of the total incident wave that is reflected, while MSE_T_ provides information about the fraction of the non‐transmitted wave. A more suggestive presentation of the results that gives absolute comparisons of the contributions of the individual mechanisms involves the analysis of mean power coefficients, namely absorption (MA), mean reflection (MR), and transmission (MT), which are derived from the measured scattering parameters (S‐parameters). These coefficients provide quantitative insight into the fraction of incidental electromagnetic energy that is absorbed within, reflected from, and transmitted through the material. To gain a more comprehensive understanding of the electromagnetic attenuation behavior of HGAs, the power coefficients MA, MR, and MT were evaluated as a function of peak pyrolysis temperature. This allows for a clearer interpretation of the shielding mechanism and its dependence on the structural and chemical evolution of the aerogels during thermal treatment.

Figure 4b represents the average power coefficients derived from S‐parameters for samples prepared at different peak temperatures. At lower peak temperatures (500°C and 600°C), MT remains relatively high (0.532 and 0.489, respectively), with MA and MR coefficients below 0.450. This suggests minimal energy dissipation or backscattering, consistent with the observed low conductivity and poor carbonization in these samples as discussed in Figure 1i,l. At 700°C, both MA and MR increase (0.726 and 0.239, respectively), while MT significantly drops to 0.034, indicating that electromagnetic energy is primarily attenuated through absorption mechanisms such as polarization loss within the aerogel. A comparison of the nitrogen configurations for the 600 and 700°C samples reveals no significant change in the pyrrolic and pyridinic components, which act as polarization centers. Thus, the reduction in transmission between these samples is primarily attributed to the massive increase in electrical conductivity of the carbon framework from 0.190 mS m^−^^1^ at 600°C to 0.11 S m^−^^1^ at 700°C. At higher peak pyrolysis temperatures (800 and 900°C), MT decreases further to below 0.001, while the MR rises to 0.429 and 0.717, respectively. The pyrrolic and pyridinic nitrogen contents at these temperatures remain comparable to those at 700°C, indicating a similar density of polarization centers. This suggests that the increase in attenuation primarily arises from the substantial rise in electrical conductivity due to progressive carbonization and graphitic domain formation at 900°C, rather than from changes in nitrogen configuration, which remain relatively consistent. Figure S7d–f presents the power coefficients of the samples as a function of frequency. The transmission coefficient (T) decreases significantly at the peak temperature of 900°C compared to lower temperatures, with this reduction remaining consistent across the entire frequency range. Similarly, the reflection (R) and absorption (A) coefficients don't exhibit drastic variation with frequency, indicating weak frequency dependence. The impedance mismatch between the highly conductive aerogel and free space enhances interfacial reflection, particularly at the highest carbonization temperature (900°C). Overall, these results demonstrate that thermal processing governs a gradual transition in the dominant attenuation mechanism: from high transmission to absorption‐dominated materials at intermediate temperatures, and finally to reflection‐dominated materials at high temperatures. This mechanistic understanding is crucial for the rational design of HGA‐based EMI shielding materials tailored for specific application requirements, whether targeting internal loss mechanisms or surface reflection.

The EMI shielding performance of a material is highly influenced by its structural morphology and electrical properties, and as discussed in Figure 2, for HGA, these attributes could be tuned by controlling the heating rate. To investigate the influence of heating rate on EMI shielding behavior, HGA samples were synthesized at 5, 15, 25, and 35°C/min, maintaining a fixed peak pyrolysis temperature of 900°C. The MSE was evaluated over the X‐band frequency range (8–12 GHz) using samples of thickness 5 mm, as presented in Figure 4c. A decreasing trend in MSE_T_ is observed with increasing heating rate: the sample prepared at 5°C/min exhibits the highest MSE_T_ of 38.74 dB, whereas samples carbonized at 15, 25, and 35°C/min display progressively lower MSE_T_ values of 23.49, 22.08, and 18.42 dB, respectively. Although all samples are expected to display similar degrees of graphitization, this divergence in shielding performance may be attributed to microstructural differences [16]. Slower heating promotes gradual decomposition and stabilization of the carbon network during the foaming stage, resulting in more consolidated and moderately dense architectures with low surface area and high electrical conductivity (Figure S4; Figure 2l). In contrast, higher heating rates lead to rapid gas evolution and expansion, resulting in aerogels with ultralow density, high surface area, and increased porosity (Figure 2l; Figure S4 and Table S3). As discussed in Figure 2k, there isn't a drastic difference in the pyrrolic and pyridinic components across the samples prepared at different heating rates, which act as polarization centers. The enhanced porosity reduces the effective number of conductive pathways available for charge transport and electromagnetic wave interaction. As a result, both MSE_A_ and MSE_R_ components of shielding decrease with heating rate, as shown in Figure 4c (black and red curves). The MSE_A_ drops from 33.22 dB (5°C/min) to 15.89 dB (35°C/min), while MSE_R_ declines from 5.52 to 2.53 dB. Figure S8a–c depicts the frequency response of SE, revealing minor change across the X‐band range, indicating that the shielding performance of the HGAs is largely independent of frequency. These findings indicate that the EMI attenuation behavior of HGAs is strongly correlated with their structural framework, which can be effectively modulated through pyrolysis kinetics.

To gain deeper insight into the electromagnetic shielding behavior of HGAs as a function of heating rate, we analyzed the underlying attenuation mechanisms using mean power coefficients derived from S‐parameter measurements (Figure 4d). As the heating rate is increased from 5 to 35°C/min, the MR decreases from 0.717 to 0.438, while MA rises from 0.283 to 0.548, indicating a shift in the dominant shielding mechanism from reflection to absorption. MT also increases over this range, from 1.34 × 10^−4^ to 1.44 × 10^−2^, indicating a gradual reduction in overall MSE. Figure S8d–f presents the power coefficients of the HGAs as a function of frequency. The transmission coefficient remains nearly constant across the X‐band for all heating rates, whereas the reflection and absorption coefficients show a slight change with rising frequency. This behavior can be attributed to enhanced electromagnetic scattering and multiple internal reflections within the hierarchically porous network formed at higher heating rates, where the pore size distribution becomes finer (Figure S4f) [31]. As the wavelength decreases at higher frequencies, electromagnetic waves interact more strongly with the internal HGA interfaces and air voids, increasing the number of scattering events and prolonging the propagation path within the aerogel, resulting in a gradual shift toward higher absorption.

It is well established that dense, highly conductive materials primarily attenuate electromagnetic radiation through reflection due to the large impedance mismatch between air and the shielding surface [32]. In contrast, porous conductive materials behave as effective media composed of both air and the solid matrix. The overall or effective permittivity of such porous structures can be described using the Maxwell–Garnett effective medium theory [33],

$$\varepsilon_{\mathrm{eff}}=\varepsilon_{m}\;\frac{\left(\varepsilon_{i}+2\varepsilon_{m}\right)+2f\left(\varepsilon_{i}-\varepsilon_{m}\right)}{\left(\varepsilon_{i}+2\varepsilon_{m}\right)-f\left(\varepsilon_{i}-\varepsilon_{m}\right)}$$

where, ε_eff_ is the effective permittivity of the composite medium, ε_*m*_is the permittivity of the matrix, ε_*i*_is the permittivity of the inclusion (air in the case of pores), and *f*is the volume fraction of the inclusion. According to this relation, increasing porosity decreases the effective permittivity and thereby improves impedance matching between the material and free space.

In the present work, increasing the heating rate leads to higher porosity, smaller pore size, and greater surface area, which enhance impedance matching and facilitate deeper penetration of incident electromagnetic waves into the aerogel [34]. Consequently, the MR decreases while the absorption contribution increases, as evidenced by the rise in MA and reduction in MR with increasing heating rate. This behavior indicates that HGAs prepared at higher heating rates exhibit absorption‐dominated shielding, where multiple internal reflections within the hierarchical pore network and enhanced interfacial polarization further promote electromagnetic energy dissipation.

While Raman analysis indicates comparable graphitic ordering across samples, the heating rate strongly influences porosity, surface area, and density (Figure 2l; Figure S4 and Table S3), which in turn affect wave‐material interactions [2, 16]. At slower heating rates, denser structures with more conductive networks favor reflection of waves. In contrast, faster heating promotes the development of more porous morphologies that increase the likelihood of wave penetration and subsequent absorption within the aerogel structure. Thus, heating rate provides a direct handle to modulate between reflection and absorption‐based attenuation in HGAs.

The thickness of EMI shielding material plays a pivotal role in determining its attenuation performance, as it governs the extent of interaction between the incident electromagnetic wave and the material. This geometric parameter is especially important in porous systems, where wave‐matter interactions scale with both material depth and structural complexity [35]. To investigate this relationship, HGA samples synthesized at a heating rate of 35°C/min and carbonized at 900°C were fabricated with varying thicknesses of 2.5, 4, 6, and 8 mm. The corresponding MSE in the X‐band (8–12 GHz) is presented in Figure 4e, revealing a positive correlation between sample thickness and MSE. The 2.5 mm thick sample exhibits an MSE_T_ of ∼10 dB, corresponding to around 90% attenuation of the incident electromagnetic wave. As the thickness is increased to larger values, the MSE_T_ improves substantially, reaching ∼15 dB at 4 mm, 23.5 dB at 6 mm, and exceeding 35 dB at 8 mm. This trend is consistent with the 5 mm sample shown in Figure 4c, prepared under identical conditions (heating rate of 35°C min^−^^1^ and peak temperature of 900°C), which exhibits an MSE_T_ of 18.4 dB. This increasing MSE_T_ is primarily due to a substantial rise in the MSE_A_ component of attenuation represented by the black curve in Figure 4e. As thickness increases from 2.5 to 8 mm, the MSE_A_ contribution rises from 8.10 to 32.07 dB, accounting for over 94% of the total shielding in the thickest sample. In contrast, MSE_R_ values slightly increase from 0.94 to 1.95 dB over the same thickness range. This rise in attenuation may be attributed to the extended propagation path provided by thicker samples, which facilitates increased interaction between the electromagnetic waves and the aerogel structure, leading to multiple internal reflections and scattering events that enhance energy dissipation through absorption mechanisms [2, 34]. Similar to the behavior observed at peak temperatures and heating rates, Figure S9a–c show that SE of samples with different thicknesses exhibits minimal frequency dependence across the X‐band.

To further elucidate the attenuation mechanisms in HGAs as a function of material thickness, we evaluated the corresponding mean power coefficients derived from S‐parameter measurements (Figure 4f). As the sample thickness is increased from 2.5 to 8 mm, the MT decreases sharply, falling from 0.1243 at 2.5 mm to below 0.001 for the 8 mm sample, indicating that over 99.9% of the incident electromagnetic power is attenuated in the thickest samples. With increasing sample thickness, MA remains above 0.5 in all cases, confirming absorption‐dominated attenuation across the samples. Also, MR does not show any substantial change, exhibiting only a slight increase with thickness, likely due to additional internal interfaces that contribute to reflection. Moreover, the power coefficients exhibit no substantial variation across the measured frequency range (Figure S9d,e). Overall, these results demonstrate that absorption remains the primary attenuation mechanism at all thicknesses, with only a small increase in reflection at greater thicknesses. This emphasizes the importance of optimizing material thickness to maximize absorption efficiency while limiting reflection.

Overall, the electromagnetic interference shielding performance of HGAs arises from the combined influence of their multiscale structure and intrinsic chemical composition. As illustrated in Figure 4g, the interconnected porous network facilitates extended wave propagation through multiple internal reflections, increasing the chances of energy dissipation within the material. Material conductivity contributes to conduction losses by supporting mobile charge transport, while the presence of inherent heteroatoms like nitrogen functionalities enables dipolar polarization under alternating electromagnetic fields [2]. Together, these mechanisms result in effective attenuation dominated by absorption, highlighting the role of both structure and chemistry in tailoring shielding performance.

Specific shielding effectiveness (SSE, in dB·cm^2^·g^−^^1^) provides a key practical metric for assessing the EMI shielding performance of materials while accounting for their density and geometry. SSE values were calculated by dividing the total MSE by the product of the sample's thickness and density. This parameter is especially important for lightweight applications, such as aerospace, where mass is a critical constraint. Although dense materials can exhibit high absolute SE, their large mass leads to low SSE. In contrast, the present HGA combines a good absolute MSE (∼40 dB, exceeding the ∼20 dB commercial benchmark) with ultralow density, resulting in an outstandingly higher SSE. Table S5 summarizes the SSE of samples prepared at different heating rates, considering the material's low density, a more comprehensive assessment of performance emerges. Due to increased foaming and expansion at higher heating rates, the bulk density of the aerogels decreases significantly, from 12.49 mg/cm^3^ at 5°C/min to just 2.27 mg/cm^3^ at 35°C/min. SSE increases from ∼ 6203 dB·cm^2^·g^−^^1^ at 5°C/min to ∼16203 dB·cm^2^·g^−^^1^ at 35°C/min, demonstrating that the weight‐normalized performance improves despite the decrease in MSE. This behavior highlights a fundamental trade‐off between MSE and structural lightness, where lower heating rates favor higher MSE, whereas faster heating rates promote the formation of ultralight, highly porous HGA frameworks that not only enhance SSE but also enable absorption‐dominated attenuation.

**FIGURE 5:**
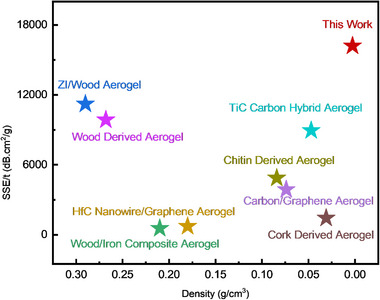
Comparison of the SSE of HGA with similar materials and those produced through more complex processing methods.

As presented in Figure 5 and Table S6, the HGAs developed in this work achieve SSE values that not only far exceed those of previously reported carbon aerogels produced through simple pyrolysis but also surpass or compete with carbon‐based materials obtained through complex multistep processes involving functionalization, chemical activation, or templating. For instance, the SSE of HGAs surpasses that of cork‐derived carbon aerogels, which were also synthesized without chemical activation or post‐synthetic modification, by more than an order of magnitude. These values represent the highest reported SSE among carbon aerogels produced through simple pyrolysis without the aid of templating, functionalization, or multi‐step processing. For context, we also benchmarked our results against more complex carbon‐based aerogels derived from graphene, CNTs, or hybrid nanomaterials that require complicated, multistep, and resource‐intensive fabrication methods. Overall, the HGAs presented here demonstrate exceptionally high SSE and structural tunability, enabling precise control over the attenuation mechanism, all achieved through simple processing. This combination of ultralow density, strong attenuation, and process simplicity positions HGAs as promising candidates for EMI shielding applications such as aerospace, as summarized in Figure S9. Overall, the HGAs presented here demonstrate exceptionally high SSE and structural tunability, enabling precise control over the attenuation mechanism, all achieved through simple processing. This combination of ultralow density, strong attenuation, and process simplicity positions HGAs as promising candidates for EMI shielding applications such as aerospace, as summarized in Figure S9.

## 3 Conclusions

In this study, we demonstrate HGA synthesized through the controlled pyrolysis of albumen, a sustainable protein‐based precursor, for high‐performance EMI shielding applications. The resulting single‐component albumen‐derived HGA displays an impressive SSE of over 16 200 dB·cm^2^·g^−^^1^ in the X‐band (8–12 GHz), surpassing previously reported similar carbon aerogels produced through simple pyrolysis. We have shown that systematic control of processing parameters, such as carbonization temperature and heating rate, together with geometric design factors like sample thickness, enables modulation of the microstructure and, consequently, control over the electromagnetic attenuation behavior. Tailoring these attributes allows absorption‐dominated shielding, which is particularly attractive for mitigating secondary electromagnetic pollution. Overall, these results establish albumen‐derived, single‐component HGAs as high‐performance EMI shielding materials obtained through a simple and tunable processing approach, offering new opportunities for sustainable applications in aerospace, electronics, and next‐generation communication technologies.

## 4 Experimental

### 4.1 Materials

Albumen protein (Good & Gather, Target Corporation, USA) was used as the precursor for graphitic aerogel synthesis. The material was used as received, without any chemical modification.

### 4.2 Synthesis of HGA

HGA is prepared by the pyrolysis of freeze‐dried albumen protein [12]. Briefly, the liquid albumen protein is first freeze‐dried, removing solvent from the sample. The freeze‐dried samples are then separately pyrolyzed at different peak temperatures of 500, 600, 700, 800, and 900°C under a nitrogen atmosphere at a heating rate of 5°C/min. Also, freeze‐dried samples are pyrolyzed at the heating rates of 1, 5, 15, 25, and 35°C/min at a peak temperature of 900°C. All the samples are held at the designated peak temperature for 4 h and then allowed to naturally cool to room temperature.

### 4.3 Characterizations

Scanning electron microscopy (SEM) was conducted using the Verios 460 XHR and FEI Quanta 200 FEG ESEM to examine the microstructure and morphology of the samples. Crystalline structure was analyzed by X‐ray diffraction (XRD) using a Bruker D8 Discover diffractometer. Fourier transform infrared (FTIR) spectroscopy was performed with a Nicolet iN10 MX spectrometer (Thermo Scientific) to assess surface functional groups. Raman spectroscopy was carried out using a Horiba spectrometer equipped with a 532 nm laser for evaluating graphitic ordering and defect structures. Surface chemical composition was investigated using X‐ray photoelectron spectroscopy (XPS/UPS) with a Thermo K‐Alpha system. TGA was performed using a TGA 8000 analyzer (PerkinElmer, USA). Nitrogen adsorption–desorption isotherms were measured at 77 K using a Micromeritics 3Flex physisorption analyzer to evaluate the specific surface area and pore structure of the samples.

The bulk density (ρ) was calculated using below equation,

$$\rho =\frac{m}{\left(l\times b\times h\right)}$$

where, *m* is the mass, and *l*, *b*, and *h* are the length, breadth, and height of the sample, respectively. The mass of each sample was measured using a high‐precision balance, and its dimensions were determined with a vernier calliper. Electrical resistance was measured using a Keithley DMM6500 digital multimeter following the standard two‐probe method. The electrical conductivity (σ) was then calculated using the equation below,

$$\sigma =\frac{L}{R\times A)}$$

where, *R* is the measured resistance, *L* is the distance between the two electrodes, and *A* is the cross‐sectional area of the sample.

The EMI shielding effectiveness was examined for the X‐band range using a waveguide method utilizing a WR‐90 RF waveguide and a Keysight N5222A PNA microwave network analyzer (See Figure S4). The aerogel monoliths were carefully cut into rectangular pieces using a sharp stainless‐steel blade to match the waveguide aperture (22.8 × 10.1 mm^2^). The cutting was performed slowly and with minimum pressure to prevent any structural deformation or damage to the samples. Scattering parameters were used to calculate absorption shielding effectiveness (SE_A_), total shielding effectiveness (SE_T_), shielding effectiveness by multiple reflections (SE_M_), and reflection shielding effectiveness (SE_R_) as per the following equations,

$$SE_{T}=SE_{R}+SE_{A}+SE_{M}$$

$$SE_{R}=-\;10log\left(1\;-\;R\right)$$

$$SE_{A}=-\;10log\frac{T}{\left(1-R\right)}$$

where,

T = Transmission Coefficient

R = Reflection Coefficient

## Author Contributions

M.S.W. and C.B.A. conceived the project. M.S.W. conducted the experiments and performed material characterization. M.S.W. and Y.O.J. carried out the EMI shielding experiments. M.S.W. and E.Z. measured sample density and electrical conductivity. M.S.W. performed the data analysis and drafted the manuscript, with support from C.B.A. P.R.P. participated in the discussions and reviewed the manuscript. All authors discussed the results and approved the final manuscript.

## Conflicts of Interest

The authors declare no conflicts of interest.

## Supporting information




**Supporting File**: smll72865‐sup‐0001‐SuppMat.docx.

## Data Availability

The data that support the findings of this study are available in the supplementary material of this article.
