# Supplementary material for: Self‐Templated Hierarchically Porous Graphitic Aerogels for Emi Shielding
Source: Small. 2026 Feb 15;22(22):e14369. doi: 10.1002/smll.202514369 (PMC13089089; doi:10.1002/smll.202514369)
Supplement: Supplementary file 1 — Supporting File: smll72865‐sup‐0001‐SuppMat.docx. [file SMLL-22-e14369-s001.docx]

**SELF-TEMPLATED HIERARCHICALLY POROUS GRAPHITIC AEROGELS FOR EMI SHIELDING**

M. Shaharyar Wani^1,2^, Yusuf O. Jimoh^3^, Erick Zaragoza^1^, Paul R. Prucnal^3^, Craig B. Arnold^1,2*^

^1^Department of Mechanical & Aerospace Engineering, Princeton University, Princeton, NJ, 08544, USA

^2^Princeton Materials Institute, Princeton University, Princeton, NJ 08544, USA

^3^Department of Electrical & Computer Engineering, Princeton University, Princeton, NJ, 08544, USA

Author: M.S.W. shaharyarwani@princeton.edu; Y.O.J. yj2827@princeton.edu; E.Z. ez6108@princeton.edu; P.R.P. prucnal@princeton.edu

^*^Corresponding Author: C.B.A. cbarnold@princeton.edu; (609)258-0250; 41 Olden Street, Princeton, NJ, 08544, USA

**Supplementary Table S1.** Elemental composition of HGAs prepared at different peak carbonization temperatures.

| Temp (°C) | C (At%) | O (At%) | N (At%) | K (At%) | Cl (At%) |
| --- | --- | --- | --- | --- | --- |
| 500 | 55.5 | 28.46 | 8.48 | 6.72 | 0.84 |
| 600 | 42.53 | 34.47 | 7.85 | 13.89 | 1.26 |
| 700 | 57.89 | 23.94 | 7.23 | 9.59 | 1.35 |
| 800 | 56.13 | 25.87 | 6.5 | 11.29 | 0.22 |
| 900 | 65.5 | 17.19 | 5.46 | 11.62 | 0.14 |


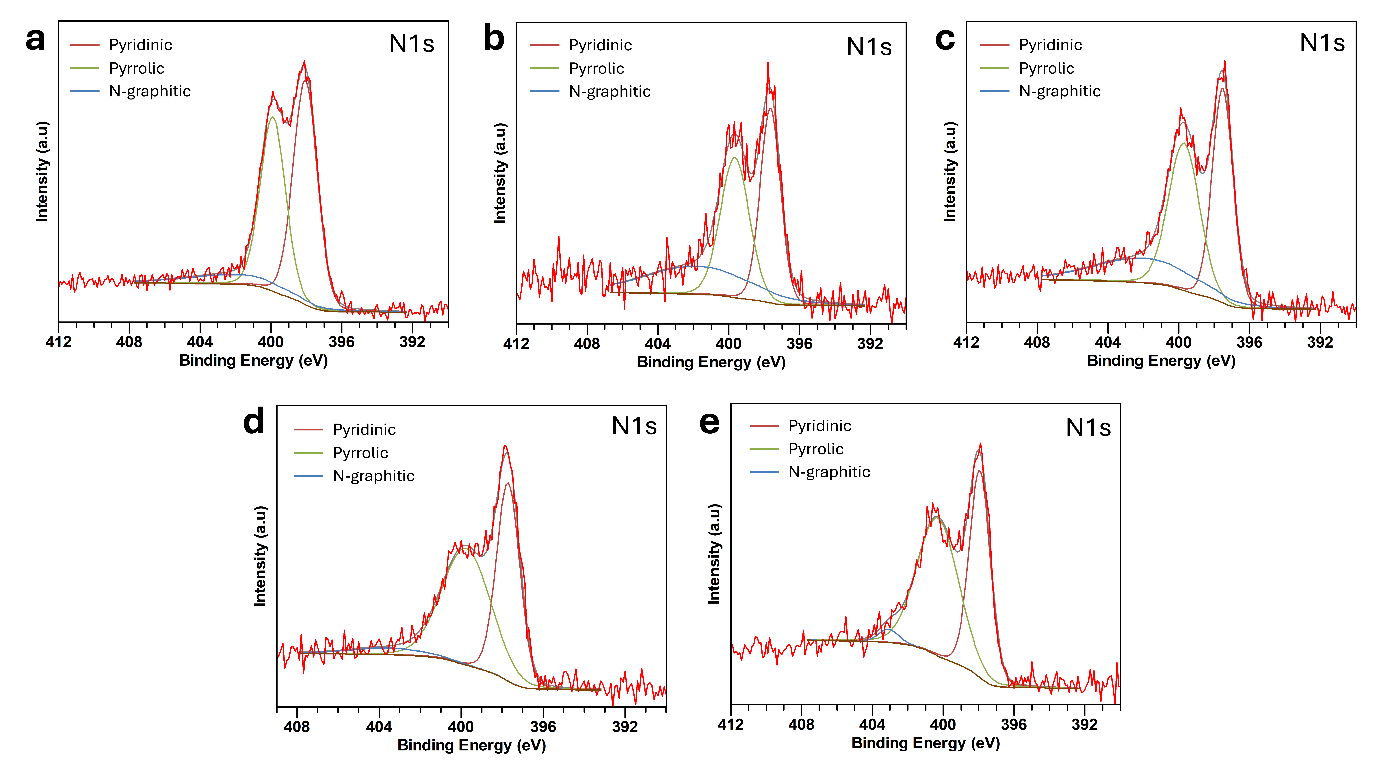


**Supplementary Figure S1.** Deconvoluted high-resolution N1s spectra of samples prepared at different peak carbonization temperatures, (a) 500 °C, (b) 600 °C, (c) 700 °C, (d) 800 °C, and (e) 900 °C.

**
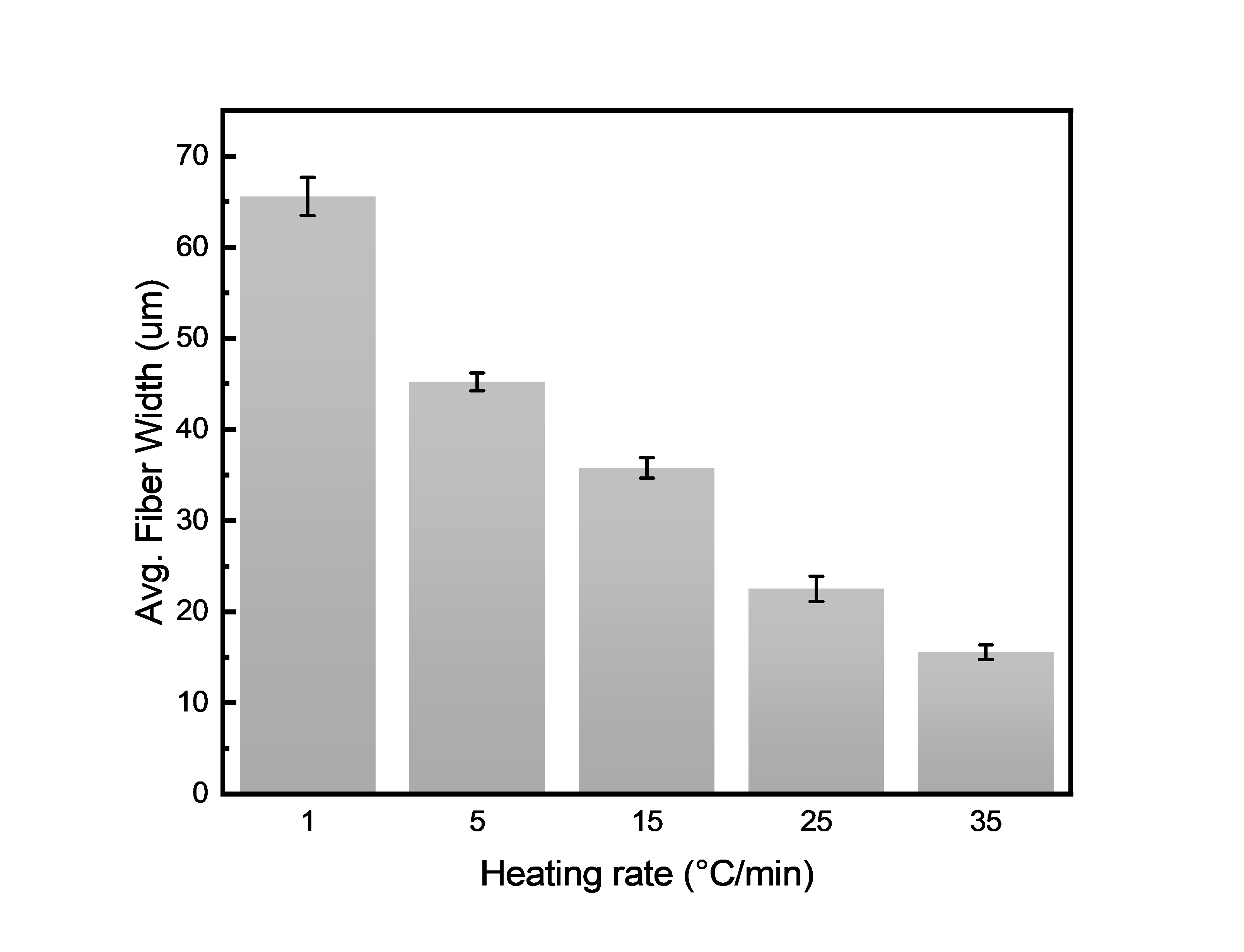
**

**Supplementary Figure S2.** Trend in fiber width as a function of heating rate.

**Supplementary Table S2.** Elemental composition of HGAs prepared at different heating rates.

| Heating Rate °C/min | C (At%) | O (At%) | N (At%) | K (At%) | Cl (At%) |
| --- | --- | --- | --- | --- | --- |
| 1 | 62.58 | 24.64 | 4.75 | 7.93 | 0.10 |
| 5 | 65.5 | 17.19 | 5.46 | 11.62 | 0.14 |
| 15 | 68.68 | 12.62 | 7.09 | 11.52 | 0.08 |
| 25 | 64.87 | 15.85 | 6.61 | 12.56 | 0.10 |
| 35 | 70.06 | 11.74 | 6.47 | 11.64 | 0.10 |


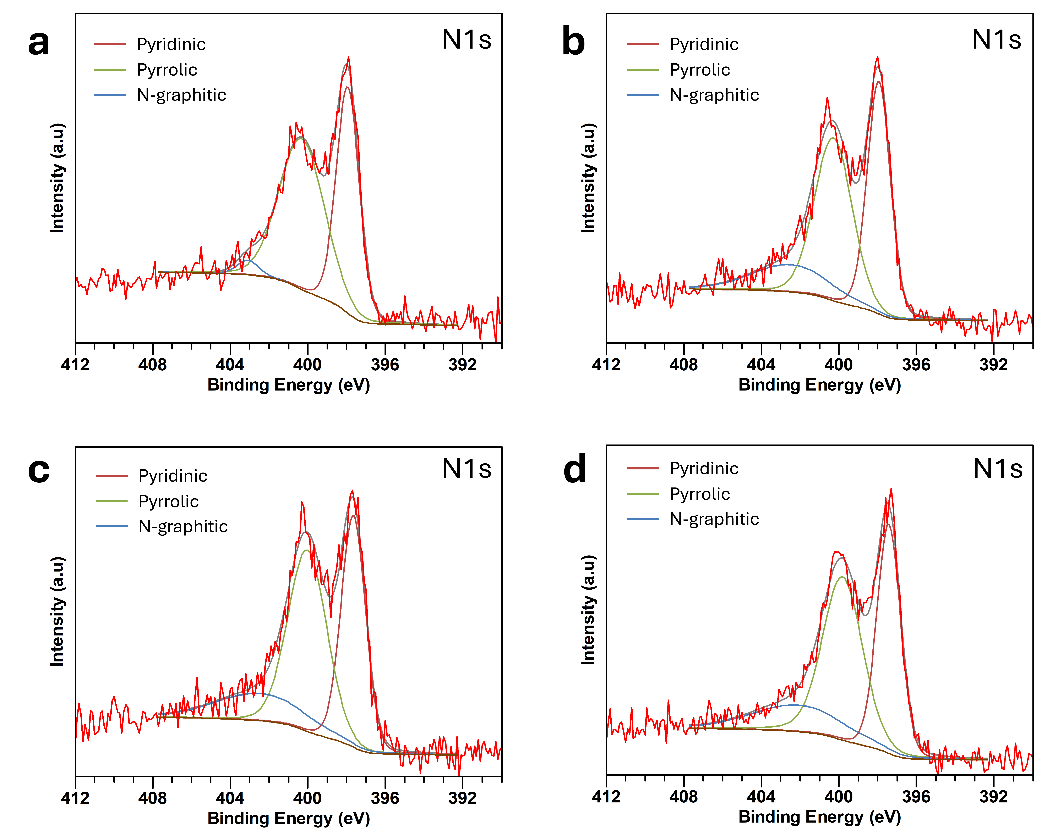


**Supplementary Figure S3.** Deconvoluted high-resolution N1s spectra of samples prepared at different heating rates, (a) 5 °C/min, (b) 15 °C/min, (c) 25 °C/min, and (d) 35 °C.


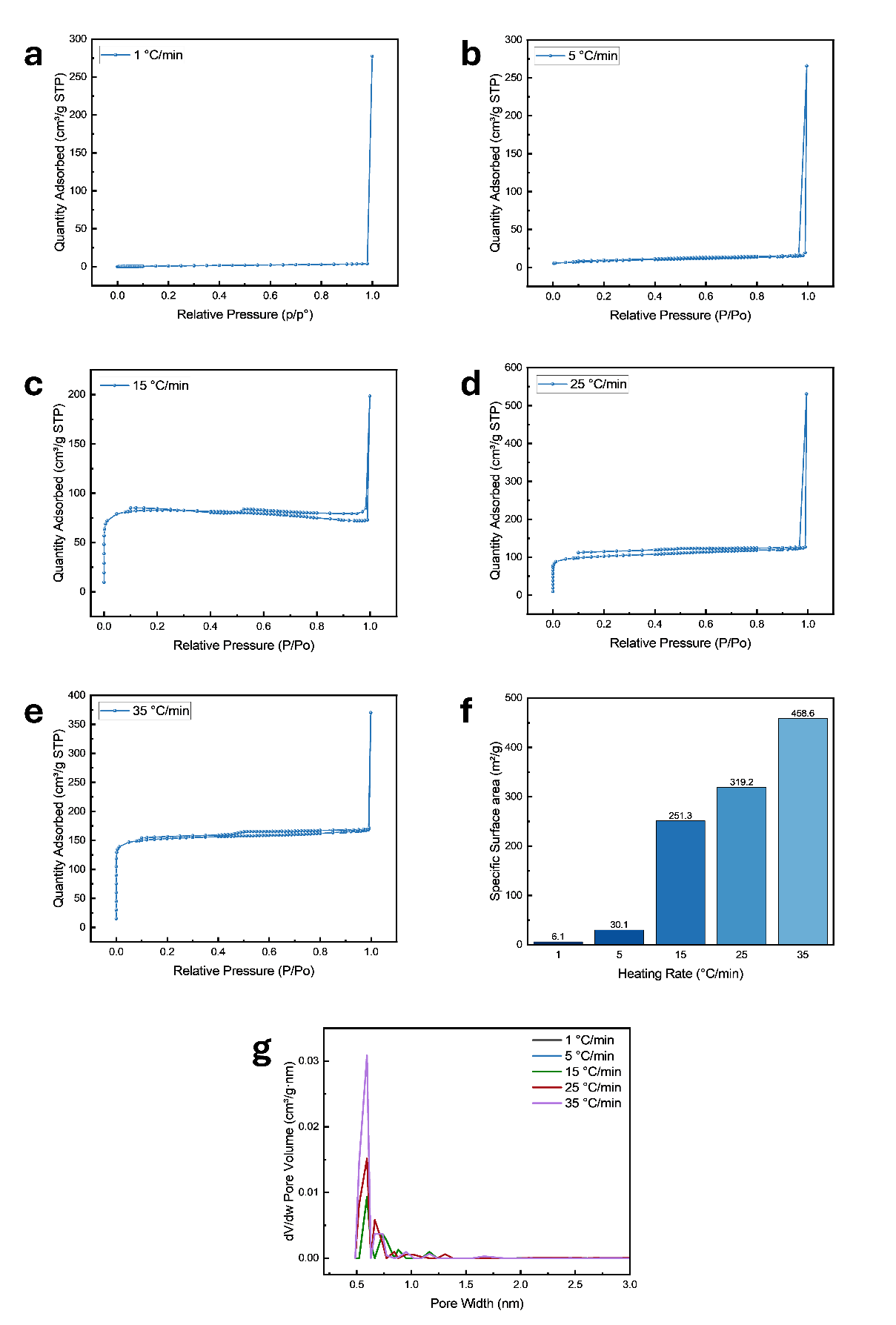


**Supplementary Figure S4.** N₂ adsorption–desorption isotherms of HGA samples prepared at different heating rates: (a) 1 °C min⁻¹, (b) 5 °C min⁻¹, (c) 15 °C min⁻¹, (d) 25 °C min⁻¹, and (e) 35 °C min⁻¹. (e) Specific surface area and (g) pore size distribution of the corresponding samples.

**Supplementary Table S3.** Porosity of HGAs prepared at different heating rates.

| Heating rate (°C/min) | Porosity (%) |
| --- | --- |
| 1 | 98.77119 |
| 5 | 99.44734 |
| 15 | 99.60405 |
| 25 | 99.7976 |
| 35 | 99.8994 |

**Supplementary Table S4.** Maximum compressive stress corresponding to 40% strain.

| Heating Rate (°C/min) | Max. Comp. Stress (kN/m^2^) | Reference |
| --- | --- | --- |
| 1.25 | 15.81 | [1] |
| 5 | 5.64 | [1] |
| 35 | 2.23 | [1] |

**
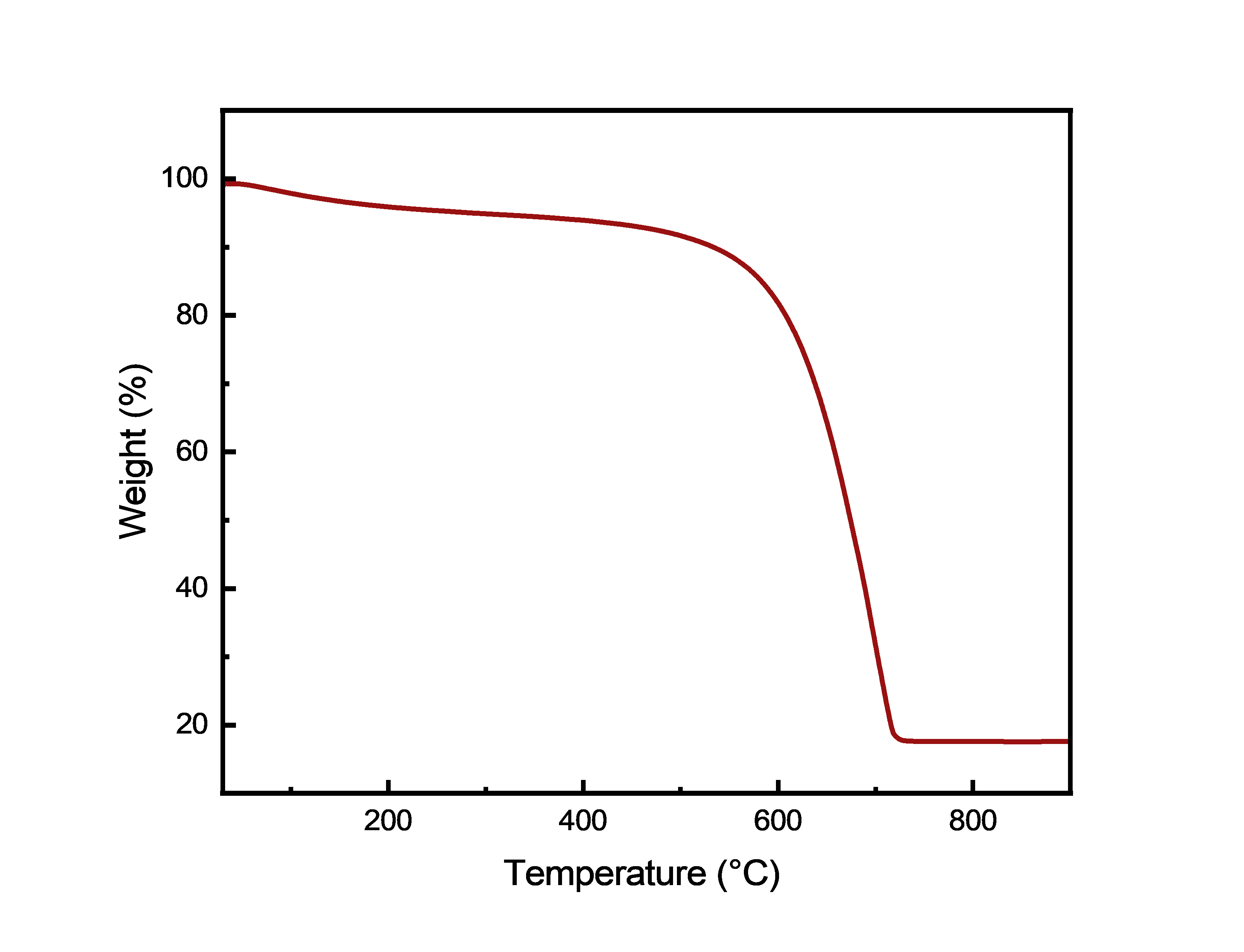
**

**Supplementary Figure S5.** Thermogravimetric analysis of HGA performed in oxidative (air) conditions.


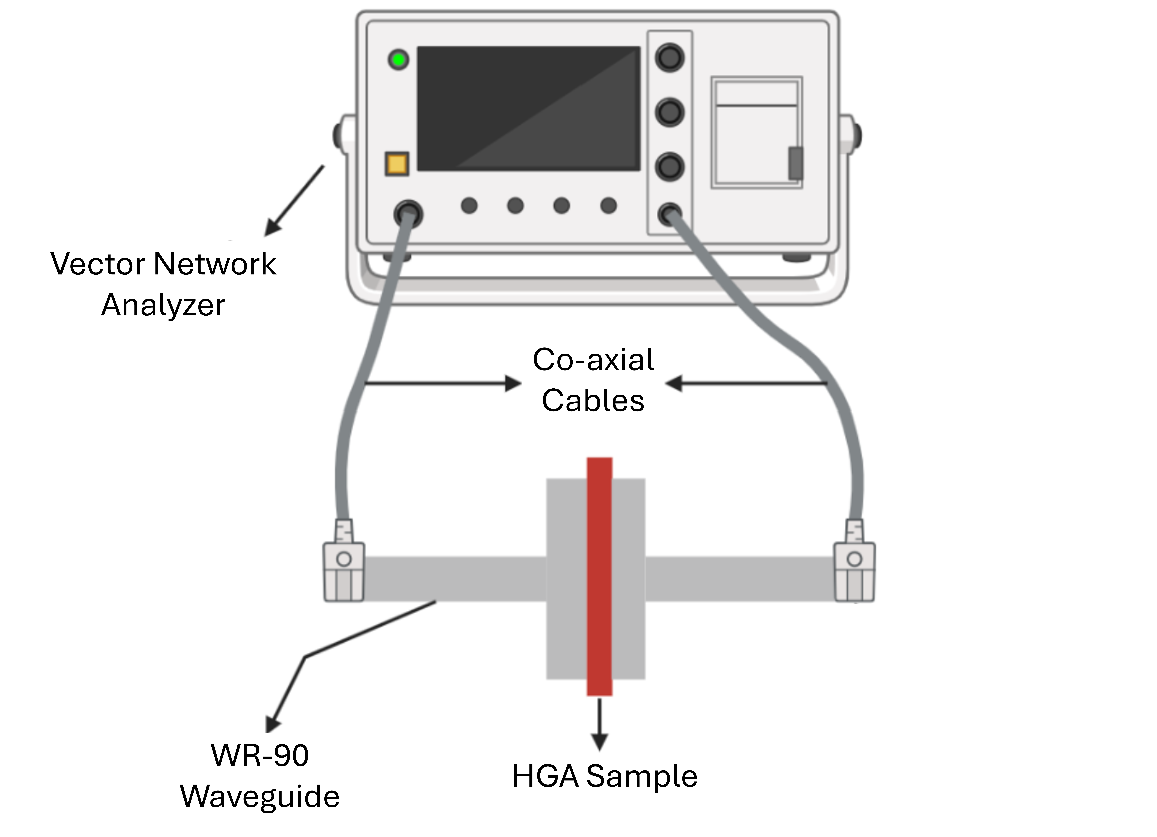


**Supplementary Figure S6.** Schematic of experimental setup for EMI shielding measurements.


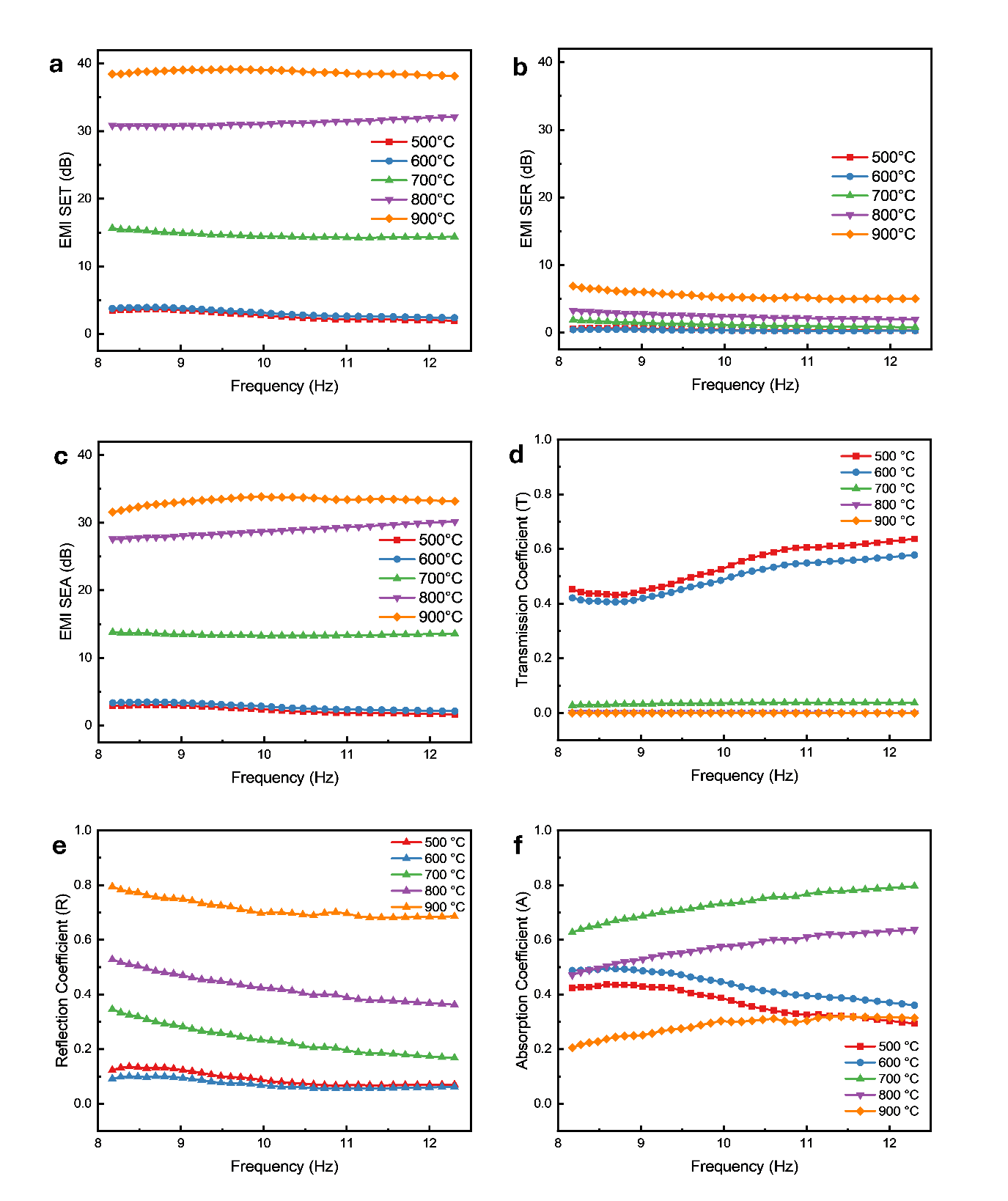


**Supplementary Figure S7.** Shielding effectiveness and power coefficients of samples prepared at carbonization temperatures of 500, 600, 700, 800, and 900 °C. (a) Total shielding effectiveness (SET), (b) reflection shielding effectiveness (SER), and (c) absorption shielding effectiveness (SEA). Corresponding power coefficients: (d) transmission coefficient (T), (e) reflection coefficient (R), and (f) absorption coefficient (A).


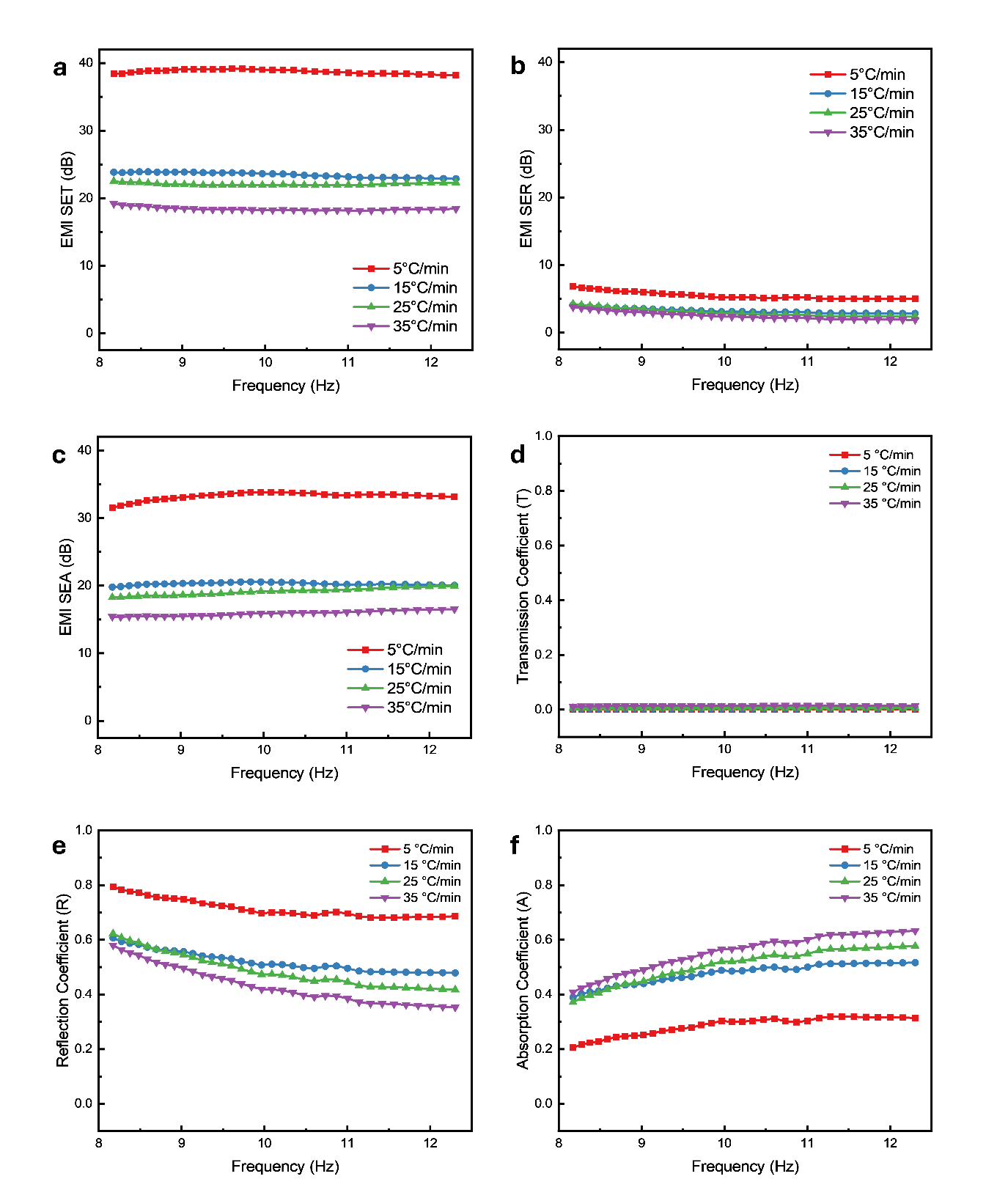


**Supplementary Figure S8.** Shielding effectiveness and power coefficients of samples prepared at heating rate of 5, 15, 25 and 35 °C/min. (a) Total shielding effectiveness (SET), (b) reflection shielding effectiveness (SER), and (c) absorption shielding effectiveness (SEA). Corresponding power coefficients: (d) transmission coefficient (T), (e) reflection coefficient (R), and (f) absorption coefficient (A).


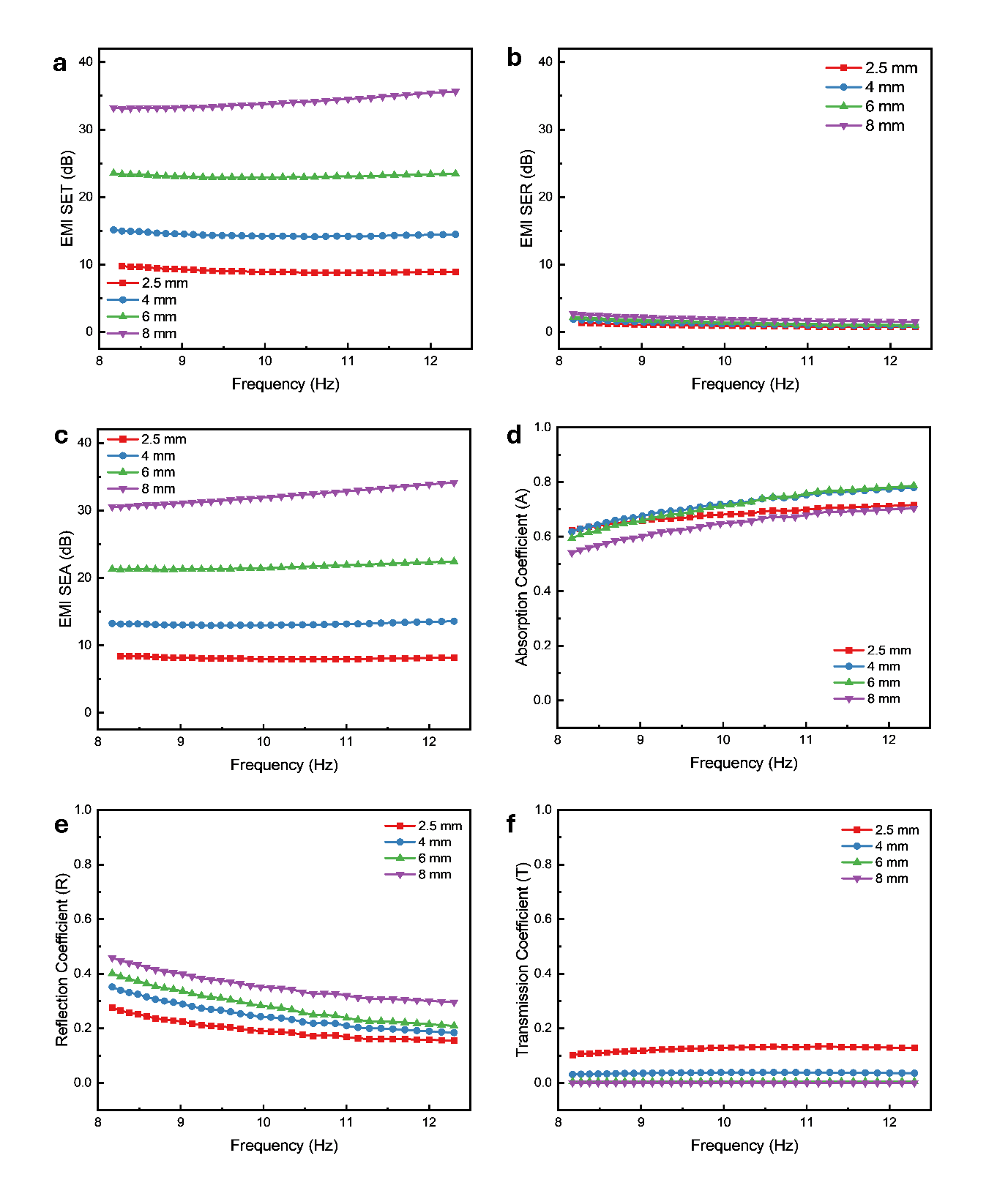


**Supplementary Figure S9.** Shielding effectiveness and power coefficients of samples prepared at a heating rate of 35 °C min⁻¹ and a peak processing temperature of 900 °C, with thicknesses of 2.5, 4, 6, and 8 mm. (a) Total shielding effectiveness (SET), (b) reflection shielding effectiveness (SER), and (c) absorption shielding effectiveness (SEA). Corresponding power coefficients: (d) transmission coefficient (T), (e) reflection coefficient (R), and (f) absorption coefficient (A).

**Supplementary Table S5.** SSE of HGAs prepared at different heating rates.

| Heating rate (°C/min) | SSE (dBcm2g-1) |
| --- | --- |
| 5 | 6203.29622 |
| 15 | 5250.62012 |
| 25 | 9601.62147 |
| 35 | 16203.31272 |

**Supplementary Table S6.** Comparison of the SSE of HGA with similar materials and those produced through more complex processing methods.

| Sample | Density (g/cm^3^) | Specific shielding effectiveness (dB.cm^2^/g) | Reference |
| --- | --- | --- | --- |
| Wood-derived Aerogel | 0.268 | 9861.41 | [2] |
| ZI/Wood Aerogel | 0.29 | 11330.04 | [2] |
| Wood/Iron Composite Aerogel | 0.21 | 551.05 | [3] |
| Carbon/Graphene Aerogel | 0.074 | 3883 | [4] |
| HfC Nanowire/Graphene Aerogel | 0.18 | 728.2 | [5] |
| TiC Carbon Hybrid Aerogel | 0.0471 | 8949.1 | [6] |
| Cork-derived Aerogel | 0.031 | 1436.05 | [7] |
| Chitin-derived Aerogel | 0.0842 | 4869.35 | [8] |
| HGA | 0.00269 | 16203.31 | This work |


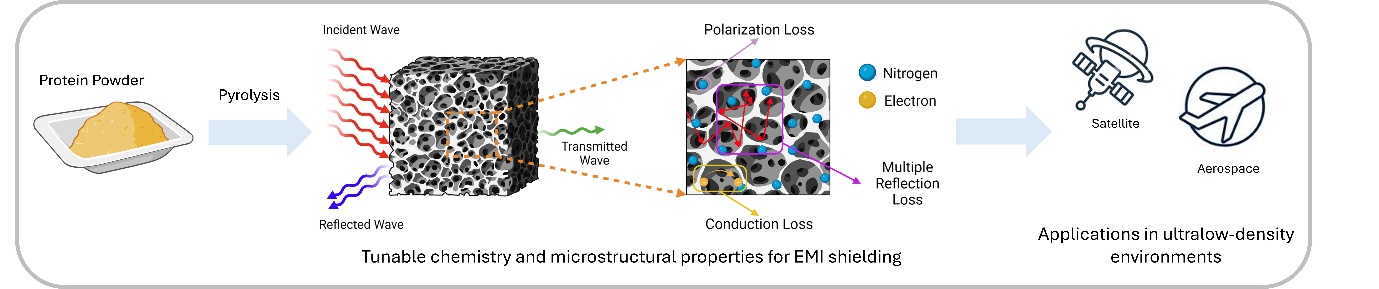


**Supplementary Figure S9.** Schematic showing HGA processing and application in EMI shielding.

**References**

[1] M.S. Wani, B. Denzer, N.J. Caggiano, R.K. Prud’homme, C.B. Arnold, Hierarchically Porous Graphitic Aerogels via Thermal Morphogenesis of Proteins for Environmental Remediation, ACS Appl. Nano Mater. 8 (2025) 8464–8472. https://doi.org/10.1021/acsanm.5c01156.

[2] X. Ma, J. Pan, H. Guo, J. Wang, C. Zhang, J. Han, Z. Lou, C. Ma, S. Jiang, K. Zhang, Ultrathin Wood-Derived Conductive Carbon Composite Film for Electromagnetic Shielding and Electric Heating Management, Adv. Funct. Mater. 33 (2023). https://doi.org/10.1002/adfm.202213431.

[3] Y. Li, S. Yan, Z. Zhang, Y. Liao, H. Rong, R. Zhao, G. Qin, Wood-Derived Porous Carbon/Iron Oxide Nanoparticle Composites for Enhanced Electromagnetic Interference Shielding, ACS Appl. Nano Mater. 5 (2022) 8537–8545. https://doi.org/10.1021/acsanm.2c01956.

[4] X. Jiang, Z. Zhao, S. Zhou, H. Zou, P. Liu, Anisotropic and Lightweight Carbon/Graphene Composite Aerogels for Efficient Thermal Insulation and Electromagnetic Interference Shielding, ACS Appl. Mater. Interfaces 14 (2022) 45844–45852. https://doi.org/10.1021/acsami.2c13000.

[5] D. Jiang, S. Tian, H. Li, Z. Du, T. Liu, D. Yan, L. Zhou, S. Bai, X. Qiang, Lightweight HfC nanowire-carbon fiber/graphene aerogel composites for high-efficiency electromagnetic interference shielding, Carbon N. Y. 219 (2024) 118788. https://doi.org/10.1016/j.carbon.2024.118788.

[6] Z. Guo, P. Ren, J. Wang, X. Hou, J. Tang, Z. Liu, Z. Chen, Y. Jin, F. Ren, Methylene blue adsorption derived thermal insulating N, S-co-doped TiC/carbon hybrid aerogel for high-efficient absorption-dominant electromagnetic interference shielding, Chemical Engineering Journal 451 (2023) 138667. https://doi.org/10.1016/j.cej.2022.138667.

[7] R.C. Pullar, R.M. Novais, A.P.F. Caetano, K.A. Krishnakumar, K.P. Surendran, Ultra-light-weight microwave X-band EMI shielding or RAM material made from sustainable pyrolysed cork templates, Nanoscale 15 (2023) 15982–15993. https://doi.org/10.1039/d3nr04411d.

[8] M.L. Wang, Z.H. Zhou, J.L. Zhu, H. Lin, K. Dai, H.D. Huang, Z.M. Li, Tunable high-performance electromagnetic interference shielding of intrinsic N-doped chitin-based carbon aerogel, Carbon N. Y. 198 (2022) 142–150. https://doi.org/10.1016/j.carbon.2022.07.016.
